# Advanced drug delivery systems for oral squamous cell carcinoma: a comprehensive review of nanotechnology-based and other innovative approaches

**DOI:** 10.3389/fddev.2025.1596964

**Published:** 2025-06-27

**Authors:** Alain Herrada Céspedes, Montserrat Reyes, Javier O. Morales

**Affiliations:** ^1^ Drug Delivery Laboratory, Department of Pharmaceutical Science and Technology, School of Chemical and Pharmaceutical Sciences, Universidad de Chile, Santiago, Chile; ^2^ Advanced Center for Chronic Diseases (ACCDiS), Santiago, Chile; ^3^ Centre of New Drugs for Hypertension and Heart Failure (CENDHY), Santiago, Chile; ^4^ Department of Oral Pathology and Medicine, Faculty of Dentistry, Universidad de Chile, Santiago, Chile

**Keywords:** oral cancer, oral squamous cell carcinoma, drug delivery systems, nanotechnology, lipid nanoparticles, intelligent hydrogels, photodynamic therapy, immunotherapy

## Abstract

Oral cancer, particularly oral squamous cell carcinoma (OSCC), poses significant challenges due to its aggressiveness, high metastatic potential, and resistance to conventional therapies. Recent advancements in drug delivery systems (DDS), including nanotechnology, intelligent hydrogels, lipid nanoparticles, and photodynamic therapy (PDT), offer innovative solutions for targeted treatment. These DDS utilize tumor-specific stimuli, such as pH variations, reactive oxygen species (ROS), and enzymatic activity, to achieve precise drug release while minimizing systemic toxicity. Cutting-edge technologies, such as microelectromechanical systems (MEMS) and artificial intelligence (AI), are enhancing the precision and personalization of DDS. Combination therapies integrating chemotherapy, PDT, and immunotherapy show promise in overcoming current limitations. Despite significant progress, challenges remain in scalability, patient-specific customization, and safety assessments. This review synthesizes the state-of-the-art in DDS for OSCC, highlighting future directions and the need for interdisciplinary collaboration to improve therapeutic outcomes and patient quality of life.

## Graphical Abstract

**Figure:**
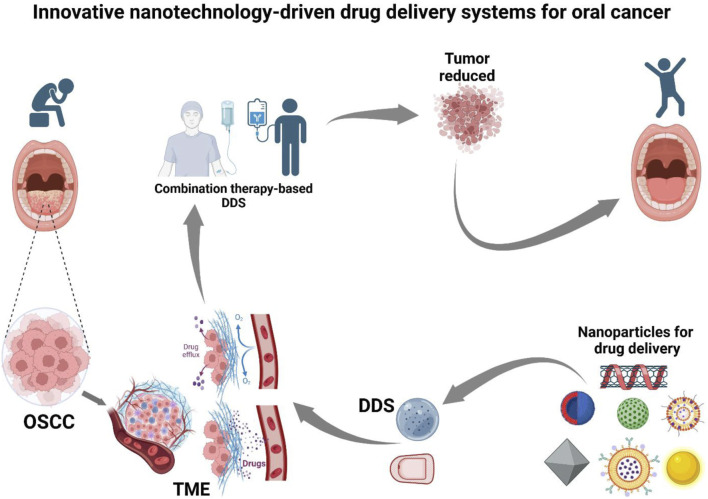
Created in BioRender. Sanchez (2025) https://BioRender.com/t13g675.

## 1 Introduction

Oral cancer, particularly OSCC, represents a significant challenge in head and neck oncology, characterized by aggressive behavior, complex pathophysiology, and frequent resistance to established therapies (Wang et al., 2015; Miranda-Filho and Bray, 2020). Originating from oral cavity epithelial cells, OSCC tends towards local invasion and lymphatic dissemination. Its development correlates strongly with tobacco use and excessive alcohol consumption, which synergistically promote carcinogenesis through DNA damage, impaired immune surveillance, and a pro-inflammatory tumor microenvironment (Li et al., 2023; Coletta et al., 2024). Human papillomavirus (HPV) infection and poor oral hygiene also contribute to its multifactorial etiology. This interplay, alongside molecular and microenvironmental changes (e.g., TP53 mutations, RAS aberrations, epigenetic modifications, dysregulated EGFR/PI3K/Akt/Wnt pathways), culminates in uncontrolled proliferation, angiogenesis, metastasis, and immune evasion (Sathish et al., 2014; Jiang et al., 2019; Yang et al., 2023).

The pathophysiology of OSCC involves genetic mutations, notably in TP53 and oncogenes like RAS, disrupting cellular proliferation-apoptosis balance and favoring tumor growth (Puteri et al., 2022). Epigenetic modifications, including DNA methylation and histone alterations, exacerbate progression by silencing tumor suppressor genes or activating oncogenic pathways. Dysregulation of critical signaling pathways (e.g., EGFR, PI3K/Akt, Wnt/β-catenin) further promotes tumor cell survival, angiogenesis, and metastatic potential (Alexander-Bryant et al., 2017; Padala et al., 2017).

The tumor microenvironment (TME) is pivotal in OSCC progression. Composed of cancer-associated fibroblasts, immune cells, endothelial cells, and an extracellular matrix, it fosters a pro-tumorigenic landscape rich in cytokines, growth factors, and matrix-remodeling enzymes, contributing to immune suppression, angiogenesis, and drug resistance (Shan et al., 2021). Hypoxia, a hallmark of solid tumors, further drives malignant behavior by upregulating hypoxia-inducible factors (HIFs), enhancing glycolysis, and promoting angiogenesis (Kim et al., 2023).

Physiologically, the oral cavity poses unique challenges for cancer therapy due to its high vascularity, continuous epithelial cell turnover, and saliva, which can impede effective drug delivery (Lim et al., 2021). Saliva can prematurely degrade or dilute therapeutic agents, reducing bioavailability. Furthermore, the dense extracellular matrix (ECM) and interstitial fluid pressure within OSCC tumors obstruct drug penetration, diminishing efficacy (Netti et al., 2000).

Current OSCC treatment involves primary surgical excision with or without neck dissection and radiotherapy, with chemotherapy reserved for advanced stages. However, standard chemotherapy is constrained by systemic toxicity, inadequate tumor selectivity, and multidrug resistance (MDR) (Li et al., 2023). These limitations significantly impact outcomes, leading to suboptimal responses, high morbidity, and mortality. Despite advancements, local recurrence, regional metastasis, and systemic side effects remain major obstacles to improving patient prognosis and quality of life (Yang et al., 2020; Shetty et al., 2022).

These unmet clinical needs underscore the urgent impetus for innovative strategies specifically targeting OSCC’s molecular and physiological characteristics while minimizing adverse effects (Asar et al., 2019). Advanced DDS, particularly those leveraging nanotechnology, have emerged as promising solutions to revolutionize OSCC treatment (Golla et al., 2013; Nguyen and Hiorth, 2015; Ceron Jayme, Ferreira Pires and Tedesco, 2020). The growing attention on DDS for oral cancer stems from their potential to overcome conventional therapy shortcomings by enabling targeted delivery, controlled release, enhanced drug accumulation in malignant tissues, reduced systemic toxicity, and potential bypass of drug resistance (Saiyin et al., 2014; Li et al., 2020).

Polymersomes, for instance, show significant potential as nanocarriers, effectively encapsulating chemotherapeutic agents like doxorubicin (DOX) and paclitaxel. These synthetic vesicles facilitate controlled drug release in the acidic and hypoxic OSCC microenvironment, enhancing therapeutic precision (Colley et al., 2012). Similarly, nanocarriers functionalized with hyaluronic acid effectively target CD44 receptors, often overexpressed in OSCC, achieving heightened drug specificity and accumulation at tumor sites (Pornpitchanarong et al., 2020).

Recent advancements in “smart” drug delivery systems, including thermosensitive hydrogels and stimuli-responsive nanoparticles, have broadened the therapeutic repertoire. These systems capitalize on pathological features such as pH gradients, enzymatic activity, and hypoxia to ensure site-specific drug release, improving efficacy while reducing systemic toxicity (Li et al., 2012; 2022). Additionally, cell membrane-coated nanoparticles mimic natural cell membranes, enhancing biocompatibility and evading immune clearance (Shi et al., 2024).

This comprehensive narrative review synthesizes the state-of-the-art in innovative DDS for OSCC, exploring nanotechnology-based and other advanced strategies. By integrating cutting-edge technologies, including stimuli-responsive systems, localized therapies, and emerging tools such as MEMS and AI, with a comprehensive understanding of OSCC biology, these innovative platforms offer a promising pathway toward more precise, effective, and personalized therapeutic approaches. Furthermore, this review critically analyzes recent developments, highlighting both significant progress and remaining barriers like scalability, safety, and patient-specific customization, in the quest for optimal therapeutic strategies to improve clinical outcomes and patient quality of life in oral cancer.

## 2 Materials and methods

### 2.1 Search strategy

A structured literature search was performed using PubMed, Scopus, Embase, and Web of Science for studies published between 1 January 2000, and 31 March 2025. A combination of Medical Subject Headings (MeSH) and free-text terms ensured comprehensive coverage. The search query included: (“oral squamous cell carcinoma” OR “oral cancer”) AND (“nanoparticle” OR “drug delivery system” OR “DDS”) AND (“stimuli-responsive” OR “microelectromechanical systems” OR “MEMS” OR “artificial intelligence” OR “AI” OR “localized therapy”). Boolean operators and field-specific filters were applied to refine results.

### 2.2 Study selection and eligibility criteria

Retrieved records were imported into Zotero for reference management and de-duplication. Articles were initially screened by title and abstract, followed by full-text analysis for eligibility based on predefined inclusion criteria:

a) Focus on innovative DDS specifically for OSCC treatment.
b) Reporting preclinical or clinical data (*in vitro*, *in vivo*, or early-phase clinical trial results) relevant to DDS performance.
c) Involving advanced or emerging DDS technologies (stimuli-responsive platforms, MEMS-integrated systems, AI-guided delivery, or localized drug administration).

Exclusion criteria included:

a) Review articles, editorials, conference abstracts, or opinion pieces lacking original experimental data.
b) Reports without sufficient methodological or technical detail to assess the DDS contribution.

Articles were also excluded if they lacked original data, were irrelevant to OSCC DDS, or did not provide sufficient methodological information for scientific interpretation.

## 3 Nanotechnology in drug delivery for oral cancer

Nanotechnology has emerged as a pivotal strategy in evolving drug delivery systems, particularly for OSCC. This aggressive malignancy often resists traditional therapies, which suffer from poor specificity, systemic toxicity, and limited bioavailability, compromising efficacy and exacerbating patient suffering (Chaughule, 2018; Lim et al., 2021). Nanoparticles, with their unique physicochemical properties, offer a robust solution by enabling precise targeting of malignant cells, enhancing drug stability, and significantly reducing collateral damage to healthy tissues (Shi et al., 2024). However, successful nanotechnology application in OSCC requires an in-depth understanding of the tumor’s distinct biological characteristics, including its microenvironment, heterogeneity, and complex immune interactions. This foundational knowledge is indispensable for designing efficient nanocarriers capable of navigating OSCC’s inherent biological barriers, ensuring effective drug delivery to the tumor site (Sah et al., 2018).

### 3.1 Polymeric nanoparticles in the treatment of oral cancer

Polymeric nanoparticles have shown significant potential in overcoming limitations of conventional chemotherapy by encapsulating agents such as docetaxel and paclitaxel (Sartaj et al., 2021), enabling sustained and controlled drug release at the tumor site. This approach is especially relevant in OSCC, where localized, prolonged delivery helps maintain therapeutic concentrations in the tumor while minimizing exposure to healthy oral mucosa, thereby reducing local and systemic toxicity.

Among the polymers explored, poly (lactic-co-glycolic acid) (PLGA) has gained particular interest due to its biocompatibility, biodegradability, and capacity for prolonged drug action (Saiyin et al., 2014). PLGA formulations protect encapsulated drugs from premature degradation, ensuring they remain active until reaching the target site.

Despite these advantages, achieving clinical success with PLGA nanoparticles hinges on maintaining a delicate balance between controlled release kinetics and the prevention of premature polymer degradation. While rapid degradation of the polymer may facilitate drug release, it also risks compromising the formulation’s long-term stability and reducing drug retention at the tumor site. To mitigate these challenges, advancements in polymer engineering, including the development of copolymer blends and the incorporation of lipophilic drugs, are focused on enhancing encapsulation efficiency and targeting specificity (Endo et al., 2013; Saiyin et al., 2014; Ceron Jayme, Ferreira Pires and Tedesco, 2020). Preclinical studies in OSCC models highlight the importance of tailoring degradation and release profiles to the tumor microenvironment.

#### 3.1.1 Controlled release and stability in the bloodstream

The controlled release of drugs from nanoparticles is essential for maintaining therapeutic concentrations over time, especially for agents with limited stability in physiological environments. Polymeric carriers like PLGA protect drugs from premature degradation, ensuring active compounds remain potent until reaching the target site (Gupta et al., 2018). However, achieving sustained release without compromising stability remains challenging. Rapid polymer degradation may trigger premature release and reduce tumor-site retention, whereas slower degradation can delay therapeutic action.

The stability and clearance of nanoparticles in the bloodstream are significantly affected by immune recognition, which can lead to rapid elimination and reduced circulation time (Tian et al., 2022). To address this, PEGylation (polyethylene glycol conjugation) to the nanoparticle surface, is widely employed. This hydrophilic coating helps shield nanoparticles from immune detection, prolonging systemic circulation. Numerous studies have shown that PEGylation reduces immune uptake, enhancing nanoparticle stability and half-life. However, emerging evidence indicates that PEGylation may impair nanoparticle internalization by tumor cells, potentially limiting therapeutic efficacy (Suk et al., 2016). This has prompted the development of advanced delivery systems capable of responding to tumor microenvironmental cues, such as pH or enzymatic activity, to trigger drug release selectively at the tumor site. (Kang et al., 2020).

Ensuring nanoparticle stability in the bloodstream is critical for systemic administration, particularly in advanced or metastatic OSCC. Polymeric nanoparticles must circulate sufficiently to reach the tumor and accumulate via mechanisms such as the EPR effect or active targeting (Vagena et al., 2025). As alternatives to PEGylation, zwitterionic polymers are being explored for their ability to reduce immunogenicity and minimize interactions with plasma proteins that may provoke immune responses. Recent studies highlight their potential to enhance bioavailability and improve targeting selectivity in tumor therapy (Zheng N. et al., 2019; Peng et al., 2020).

#### 3.1.2 Enhanced permeability and retention (EPR) effect

The EPR effect is a key mechanism in nanoparticle-based drug delivery, enabling preferential accumulation in solid tumors due to their leaky vasculature and poor lymphatic drainage. While this effect supports targeted delivery in many cancers, its efficacy in OSCC is hindered by the dense, fibrotic extracellular matrix (ECM), which restricts nanoparticle penetration and limits the full therapeutic potential of EPR-mediated accumulation (Shi et al., 2020).

Recent developments in nanoparticle design aim to overcome the limitations posed by the dense ECM in OSCC by modifying surface properties such as charge and hydrophilicity to improve tissue penetration (Cahn et al., 2024). These changes enhance nanoparticle diffusion through the tumor interstitium, increasing the likelihood of reaching cancer cells. Additionally, functionalization with enzymes like collagenase has proven effective in degrading ECM components, allowing deeper tumor infiltration without harming adjacent healthy tissues. This dual approach—surface modification and enzymatic degradation—significantly improves drug distribution and therapeutic outcomes (Chen M. et al., 2023). Enhancing nanoparticle penetration through the fibrotic OSCC ECM is essential to complement the passive targeting achieved by the EPR effect.

Despite these advancements, a major challenge remains the variability of the EPR effect across different patients due to tumor heterogeneity. The EPR effect is not uniform across all tumors or patients, as variations in vascular permeability, ECM composition, and other microenvironmental factors can significantly impact nanoparticle accumulation. This variability underscores the need for more individualized approaches to nanoparticle drug delivery.

To overcome the limitations of the EPR effect in OSCC, researchers are employing combination strategies that integrate passive targeting with active mechanisms. Active targeting involves functionalizing nanoparticles with ligands that bind to receptors overexpressed on tumor cells, enabling more selective drug delivery. By coupling EPR-based accumulation with receptor-mediated targeting, this dual strategy enhances nanoparticle localization and specificity, offering a more effective and tailored approach to drug delivery in OSCC (Kang et al., 2020).

#### 3.1.3 Biocompatibility and biodegradability

Biodegradable polymers such as PLGA are widely used in DDS due to their excellent biocompatibility, reducing the risk of long-term toxicity and making them suitable for repeated or localized administration in OSCC treatment. This is particularly important for delivery systems applied within the sensitive oral cavity. However, balancing biocompatibility with sustained drug release remains a challenge. Rapid PLGA degradation may compromise drug retention, undermining the prolonged therapeutic effect needed for optimal treatment outcomes.

To overcome the limitations of rapid polymer degradation, dual-polymer systems combining PLGA with polymers like polycaprolactone (PCL) or PEG have been widely explored (Gupta et al., 2018). These combinations enhance drug retention while maintaining biocompatibility and minimizing adverse effects. PEG, in particular, is extensively used for its capacity to prolong circulation time by protecting nanoparticles from immune recognition and clearance. Its utility in long-circulating DDS has made it a staple in nanoparticle engineering. However, growing evidence highlights potential concerns related to PEGylation. Prolonged exposure can induce the formation of anti-PEG antibodies—a phenomenon known as the “PEG dilemma”—which may lead to immune responses, faster nanoparticle clearance, and reduced therapeutic efficacy (Yin et al., 2023). Additionally, PEGylation can influence drug release kinetics, as the PEG shell may act as a barrier, impeding controlled drug diffusion and compromising overall treatment performance.

To address these challenges, alternative strategies such as zwitterionic polymers and hyperbranched polyglycerols (HPGs) are being investigated for their ability to provide prolonged circulation without provoking significant immune responses. These materials exhibit ultralow fouling characteristics, limiting protein adsorption and immune recognition while supporting efficient drug delivery (Deng et al., 2014; Peng et al., 2020). Additionally, stimuli-responsive PEG derivatives that degrade or detach under tumor-specific conditions—such as low pH or enzymatic activity—are being developed to improve site-specific drug release and overcome issues related to conventional PEGylation (Liang et al., 2021). Continued research is essential to refine these dual-polymer and PEG-alternative systems, ensuring the preservation of circulation time and release control while minimizing immunogenicity. This includes elucidating the mechanisms of anti-PEG antibody formation and creating predictive models for long-term safety. Such advances will be critical for fully realizing the therapeutic potential of PLGA-based and hybrid polymeric delivery systems (Duskey et al., 2020).

#### 3.1.4 Active targeting capabilities

Active targeting represents a key advancement in nanoparticle-based drug delivery for OSCC, relying on the functionalization of nanoparticles with ligands that bind selectively to receptors overexpressed in tumor cells, such as CD44 and folate receptors. This strategy enhances drug specificity, minimizes systemic toxicity, and reduces off-target effects by directing therapeutic agents to malignant tissues, including both primary and metastatic OSCC lesions.

Hyaluronic acid (HA), a natural CD44 ligand with high affinity and excellent biocompatibility, is among the most studied molecules for this purpose (Diao et al., 2019). Due to the frequent overexpression of CD44 in OSCC, particularly in tumor-initiating cells, HA-functionalized polymeric nanoparticles offer a promising platform. Studies have demonstrated that HA-coated nanoparticles significantly increase cellular uptake in CD44-overexpressing OSCC cells. De Capua et al. (2021) reported a fourfold enhancement in drug accumulation using HA-functionalized nanoparticles compared to non-targeted formulations, leading to improved therapeutic efficacy. Additionally, catechol-functionalized nanoparticles have shown potential for precise CD44 binding and sustained drug release in OSCC models, further supporting the versatility of ligand-mediated targeting approaches (De Capua et al., 2021).

Despite these advances, challenges remain. Receptor saturation and shedding can limit the uptake and binding efficiency of functionalized nanoparticles. To overcome these, dual-targeting systems, such as combining folic acid (FA) with tumor-specific peptides, show promise in addressing receptor heterogeneity and enhancing binding across diverse tumor subpopulations (Yin et al., 2023). This dual-ligand approach provides a synergistic effect, increasing the likelihood of effective binding even in heterogeneous tumors.

Furthermore, stimuli-responsive nanoparticles, particularly pH-sensitive designs, add specificity. The acidic tumor microenvironment (pH ∼ 6.5–6.8) enables targeted drug release, complementing receptor-mediated targeting. Liu et al. (2018) reported that FA-functionalized pH-sensitive nanoparticles increased drug release and doubled tumor cell apoptosis in acidic conditions. Similarly, Chen H. et al. (2020) developed a dual-targeting system combining pH sensitivity and receptor targeting, demonstrating enhanced specificity and efficacy in preclinical OSCC models (Liu et al., 2018; Chen H. et al., 2020). The increasing reports of polymeric nanoparticles for active targeting in OSCC or HNSCC models highlight their translational potential.

#### 3.1.5 Applications in PDT

PDT, utilizing light-activated nanoparticles loaded with photosensitizers like nano-graphene oxide, shows promise as an adjunct to traditional cancer therapies (Shi et al., 2024). PDT leverages nanoparticles’ ability to generate reactive oxygen species (ROS) upon light exposure, selectively ablating tumor cells while sparing healthy tissues (Shi et al., 2018). However, light penetration into deeper tumor tissues remains a challenge, particularly in less accessible oral cavity regions. Innovations like coatings enhancing light penetration, or combining PDT with immunotherapy, are addressing these limitations. Near-infrared light-responsive nanoparticles also hold significant promise for deep tissue penetration, enabling treatment in challenging anatomical locations (Shi et al., 2018; Yang et al., 2020). Polymeric nanoparticles encapsulating photosensitizers have been explored for OSCC PDT, aiming to improve photosensitizer solubility, stability, and targeted delivery to enhance localized cytotoxic effects while minimizing systemic photosensitivity.

#### 3.1.6 Efficient tumor penetration

The dense ECM in OSCC tumors significantly hinders effective therapeutic agent delivery. Strategies like functionalizing nanoparticles with ECM-degrading enzymes or designing smaller nanoparticles (less than 100 nm) are being explored to enhance tumor penetration. Recent studies show that nanoparticles modified with ECM-modulating agents significantly improve drug delivery and distribution in preclinical OSCC models (Netti et al., 2000; Chen M. et al., 2023). Specifically, for OSCC, enhancing polymeric nanoparticle penetration through the collagen-rich ECM is critical for achieving therapeutic concentrations throughout the tumor mass. Additionally, combining nanoparticles with ultrasound-activated microbubble technology represents a new strategy to enhance tumor penetration without compromising healthy tissues (Ingram et al., 2020).

#### 3.1.7 Potential toxicity and safety considerations

While polymeric nanoparticles generally exhibit excellent safety profiles, their functionalization and surface modifications necessitate thorough preclinical evaluation for long-term toxicity. Given the oral mucosa’s sensitivity, comprehensive safety assessments, including local tissue reactions and systemic exposure, are crucial for polymeric DDS targeting OSCC. Future research should focus on developing modular nanoparticles with precise functionalization control to minimize systemic interactions while preserving therapeutic efficacy (Ahmad et al., 2021). Advances in computational modeling could aid in predicting nanoparticle behavior in biological systems, improving safety assessments and expediting safer, more effective therapeutic solutions (Eckmann et al., 2020).

Polymeric nanoparticles hold significant promise for OSCC treatment, offering targeted drug delivery, controlled release, and the potential to integrate multiple therapeutic modalities like active targeting and PDT (Yin et al., 2023). Despite persistent challenges—particularly immune clearance, ECM barriers, and receptor heterogeneity—ongoing nanotechnology advancements are poised to address these, paving the way for clinically viable nanoparticle-based therapies for oral cancer (Netti et al., 2000; Wang et al., 2015). Continuous refinement of nanoparticle design, alongside emerging complementary technologies, holds immense potential to revolutionize OSCC treatment paradigms, ultimately improving patient outcomes and minimizing side effects (Bhattacharya et al., 2023).

### 3.2 Inorganic nanoparticles in oral cancer treatment

**FIGURE 1:**
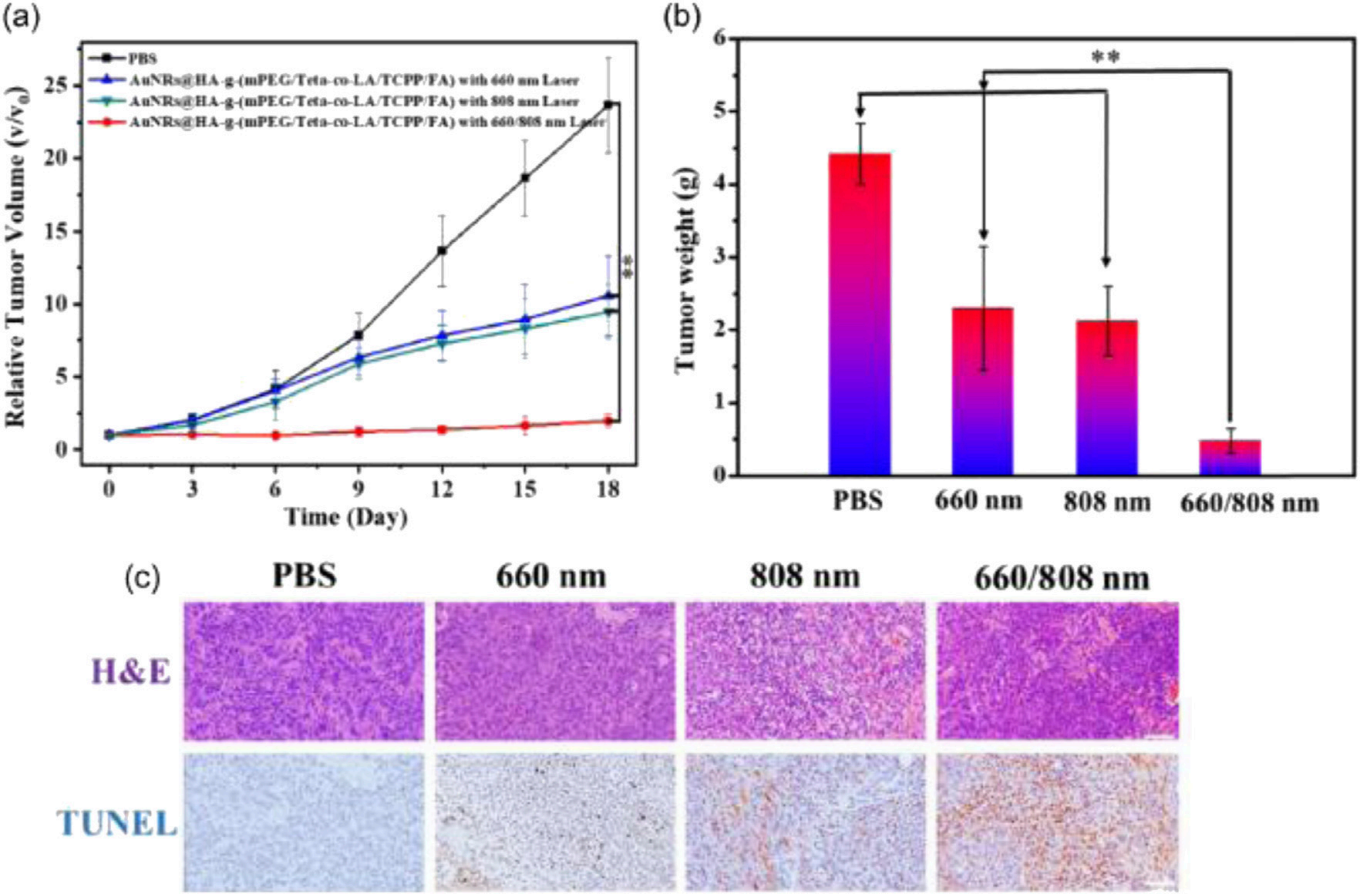
*In vivo* synergistic antitumor effect of AuNRs@HA-g-(mPEG/Teta-co-(LA/TCPP/FA) in MCF-7 tumor-bearing nude mice. **(a)** Changes of relative tumor volumes of MCF-7 tumor-bearing mice after different treatments over a period of 18 days. **p < 0.01 using Student’s t-test). **(b)** Average weights of removed tumor sections in four treatment formulations on day 18. **(c)** Representative photomicrographs of H&E and TUNEL staining analysis of isolated tumor tissues after treatment in four groups. Scale bar: 100 μm (Xu et al., 2023).

Inorganic nanoparticles have garnered significant attention as promising tools for oral cancer treatment due to their unique physicochemical properties, including small size, high surface area, and potential for surface functionalization (Tabrez et al., 2022). These attributes make them highly efficient therapeutic agent carriers, enhancing drug delivery specificity and release profiles while reducing systemic toxicity. They can overcome several biological barriers in oral cancer treatment, such as the complex tumor microenvironment, drug resistance, and limited bioavailability, Figure 1 (Xu et al., 2023). However, a deeper understanding of nanoparticle-tumor environment interactions is crucial, especially considering microenvironment variability across patients. The inherent properties of inorganic nanoparticles, like their rigidity and tunable surface chemistry, can address OSCC-specific challenges such as penetrating the dense ECM and responding to the unique TME (Saikia, 2024).

#### 3.2.1 Types of inorganic nanoparticles

##### 3.2.1.1 Gold nanoparticles (AuNPs)

AuNPs are extensively studied inorganic nanoparticles, favored for their optical properties, biocompatibility, and functionalization ease, making them ideal for oral cancer drug delivery and photothermal therapy (PTT). For OSCC, PTT with AuNPs offers a localized, non-invasive option for accessible lesions. Conjugating chemotherapeutic agents to AuNPs also enhances tumor targeting and efficacy compared to traditional methods (Abdel Hamid et al., 2021; Essawy et al., 2021). Surface functionalization, e.g., with folic acid or hyaluronic acid, improves cellular uptake in cancer cells overexpressing specific receptors, as demonstrated in preclinical OSCC models (Zhang et al., 2022). A key challenge, however, is AuNPs’ penetration of dense oral cancer ECM, necessitating optimization for deeper tumor access. Combining AuNPs with other modalities like chemotherapy or immunotherapy could boost efficacy, though improving light penetration for deeper tumors remains a significant hurdle (Asar et al., 2019; Abdel Hamid et al., 2021; Essawy et al., 2021).

##### 3.2.1.2 Titanium dioxide nanotubes (TNTs)

Represent another promising class of inorganic nanoparticles for oral cancer treatment, due to their high surface area, chemical stability, and biocompatibility. TNTs are particularly useful for sustained-release drug delivery, prolonging therapeutic effects at tumor sites and efficiently adsorbing and releasing drugs. However, clinical application is limited by insufficient long-term safety and pharmacokinetic data (Lagopati et al., 2021; Tang et al., 2023). Their behavior in the dynamic oral cancer tumor microenvironment requires further investigation, as variations in pH and biomolecules can influence drug release. More research is needed to understand TNTs’ interaction with the tumor environment and assess their clinical viability (Li et al., 2020). Emerging studies explore TNTs for drug delivery in OSCC models, highlighting their potential as controlled-release carriers within the oral cavity (Chen H. et al., 2023).

##### 3.2.1.3 Zinc oxide nanoparticles (ZnO NPs)

ZnO NPs have garnered attention for their dual antibacterial and antitumor properties, relevant for oral cancer where secondary bacterial infections complicate therapy. ZnO NPs are inexpensive, biocompatible, and easily functionalized for improved targeting. Recent studies show ZnO NPs, especially with bioactive coatings like κ-carrageenan, enhance chemotherapeutic delivery while inducing apoptosis in cancer cells (Marunganathan et al., 2024). Studies in oral cancer models investigate ZnO NPs for direct cytotoxic effects and as chemotherapy carriers, demonstrating promising antitumor activity (Wang et al., 2018). Although promising, further research is needed to assess their long-term safety, potential immune responses, and interactions within the complex oral cavity. Initial *in vivo* tolerance is good, but long-term effects, particularly with chronic use, require further investigation into clinical applicability and safety (Tabrez et al., 2022).

#### 3.2.2 EPR effect

Inorganic nanoparticles (e.g., AuNPs, mesoporous silica, magnetic nanoparticles) hold significant promise for enhancing the EPR effect in oral cancer therapy due to their high surface area and functionalization ease (Wang et al., 2017; Zuo et al., 2020). Surface modifications with polymers like PEG and specific ligands optimize tumor accumulation by exploiting both passive EPR and active targeting (Kalimuthu et al., 2018). Functionalization with enzymes such as collagenase helps overcome the dense ECM barrier in OSCC, facilitating deeper nanoparticle penetration. Despite these advancements, challenges like tumor heterogeneity, vasculature variability, and potential toxicity necessitate further refinement (Netti et al., 2000; Liu Xingguang et al., 2022). Harnessing the EPR effect with inorganic nanoparticles is a key passive targeting strategy for OSCC solid tumors, requiring overcoming the dense ECM. Combining EPR-based strategies with receptor-mediated targeting and therapeutic modalities like photothermal therapy can maximize efficacy while minimizing systemic side effects, paving the way for personalized cancer therapies (Li et al., 2023).

#### 3.2.3 Biocompatibility and biodegradability

Biocompatibility and biodegradability are crucial for the clinical application of inorganic nanoparticles, directly impacting safety and long-term efficacy. Gold nanoparticles, TNTs, and ZnO NPs exhibit high biocompatibility, minimizing adverse effects. However, concerns persist regarding the degradation of these nanoparticles into non-toxic byproducts post-therapy (Asar et al., 2019). Breakdown products, especially for TNTs and ZnO NPs, require careful study to ensure no long-term health risks (Li et al., 2012; Tabrez et al., 2022). For inorganic nanoparticles in OSCC DDS, particularly for local or repeated systemic administration, understanding their long-term fate and the toxicity of degradation products systemically and within the oral cavity is vital. While biodegradable nanoparticles are attractive for reduced long-term toxicity, balancing this with sustained drug release is challenging, as rapid degradation can lead to premature drug release and reduced efficacy. This highlights the need for research into nanoparticles with controlled degradation profiles for sustained release without compromising safety (Li et al., 2012; 2019).

#### 3.2.4 Active targeting capabilities

Active targeting with inorganic nanoparticles offers unique advantages due to their physicochemical stability and multifunctional potential, particularly valuable for OSCC treatment. Inorganic nanoparticles like AuNPs can be functionalized with ligands, such as hyaluronic acid, to specifically bind receptors overexpressed on tumor cells (e.g., CD44 in OSCC), enhancing cellular uptake and therapeutic efficacy (Oladimeji et al., 2021). These nanoparticles also offer inherent benefits, including enhanced imaging contrast and photothermal properties, exploitable for integrated diagnostic and therapeutic (theranostic) applications (Bhattacharya, 2022). Despite these attributes, challenges like receptor saturation and shedding persist, potentially limiting targeting efficiency. To overcome this, recent studies propose dual-targeting strategies incorporating multiple ligands to engage a wider array of tumor-specific markers. Furthermore, integrating active targeting with stimuli-responsive mechanisms, such as pH-triggered drug release, refines treatment specificity and precision (Alexander-Bryant et al., 2017; Li et al., 2020; Xu et al., 2023). This innovative combination leverages the distinctive properties of inorganic nanoparticles to advance targeted drug delivery in OSCC.

#### 3.2.5 Applications in PDT

PDT is an innovative cancer treatment using light-activated photosensitizers to induce cell death. Inorganic nanoparticles, such as AuNPs and TNTs, have been explored as carriers for PDT agents due to their efficient light absorption and conversion capabilities (Essawy et al., 2021; Tang et al., 2023). Recent studies indicate that combining PDT with chemotherapy can yield synergistic effects, improving tumor ablation while minimizing systemic side effects (Zhang Z. et al., 2020). Challenges persist, particularly with light penetration in deeper tumor tissues, a significant issue in the oral cavity due to light attenuation (Miranda-Filho and Bray, 2020). Recent developments in near-infrared light-responsive nanoparticles offer a promising solution for deeper tissue penetration. Further research is necessary to optimize these systems and improve their efficacy in treating deep-seated tumors (Peng et al., 2022). Inorganic nanoparticles are being investigated as photosensitizer carriers for OSCC PDT, offering advantages in localized reactive oxygen species (ROS) generation upon light activation, though optimizing light penetration for deeper oral cavity tumors remains a key challenge (Wang et al., 2024; Zhang et al., 2024).

#### 3.2.6 Stability in the bloodstream

Stability in the bloodstream is crucial for nanoparticle-based drug delivery. Inorganic nanoparticles like AuNPs and ZnO NPs often face rapid immune clearance, reducing therapeutic efficacy (Kalimuthu et al., 2018). Surface modifications, such as PEGylation, commonly enhance nanoparticle stability and circulation time. However, while PEGylation improves stability, it can also hinder cellular uptake, suggesting a need for more sophisticated approaches to balance stability with effective drug delivery (Tian et al., 2022). Ensuring the stability of inorganic nanoparticles in systemic circulation is important for reaching OSCC tumors, especially for metastatic disease, and strategies like PEGylation are used to prolong their half-life and reduce rapid clearance.

#### 3.2.7 Efficient tumor penetration

Achieving effective tumor penetration remains a significant challenge for inorganic nanoparticle-based therapies in oral cancer (Li et al., 2020). The compact extracellular matrix (ECM) surrounding OSCC tumors restricts the even distribution and deep infiltration of these particles. Recent investigations indicate that tailoring the nanoparticle surface—by incorporating ECM-degrading enzymes or by reducing particle size—can markedly enhance intra-tumoral dispersion (Yang X.-Y. et al., 2022). Furthermore, adjunctive techniques like ultrasound stimulation and magnetic guidance have shown promise in promoting deeper nanoparticle infiltration (Zhu et al., 2018). Overcoming the dense ECM in OSCC is a major hurdle for inorganic nanoparticle penetration; strategies involving ECM-degrading enzymes or smaller particles are being explored to improve their distribution within oral tumors. Beyond their role in targeted drug delivery, inorganic nanoparticles are also leveraged for their multifunctional capabilities, including photothermal therapy and PDT (Shi et al., 2024). Nevertheless, challenges persist, including overcoming the dense ECM barrier, addressing inter-patient variability, and ensuring long-term biocompatibility and safety. Continued research is vital to optimize these nanoparticle systems, refine their physicochemical properties, and ultimately surmount biological barriers in oral cancer treatment. With sustained innovation and rigorous clinical validation, inorganic nanoparticles could emerge as a pivotal component in personalized therapeutic strategies for oral cancer.

#### 3.2.8 Inorganic two-dimensional (2D) nanomaterials for drug delivery

Advances in low-dimensional nanomaterials show rapid progress towards clinical oncology applications. A prominent example is 2D hexagonal boron nitride (h-BN) nanomaterials, or “white graphite.” While less explored than graphene oxide (GO), h-BN exhibits unique properties making it an excellent drug delivery candidate (Emanet et al., 2019). h-BN nanosheets possess exceptional physicochemical properties, including chemical stability, high surface-to-volume ratio, and biocompatibility, making them efficient for targeted delivery of chemotherapeutics to tumor-specific sites (Rasul et al., 2021). For instance, h-BN applications in targeted drug delivery have demonstrated controlled and efficient release in acidic tumor microenvironments, minimizing systemic toxicity (Visani De Luna et al., 2023). This capability underscores its potential as a key material for developing more effective targeted therapies for oral carcinoma, particularly when combined with photodynamic or ROS-based therapies, where synergistic interactions with the tumor environment can amplify therapeutic effects. While still emerging, 2D inorganic nanomaterials like h-BN are being explored as drug carriers in oral cancer, leveraging their unique properties for targeted delivery and potential synergistic effects with other therapies within the OSCC TME.

### 3.3 Lipid nanoparticles (LNPs) for oral cancer therapy

LNPs represent a pivotal innovation in drug delivery for oral cancer, addressing limitations of traditional chemotherapeutics like suboptimal bioavailability, systemic toxicity, and lack of tumor specificity (Nasirizadeh and Malaekeh-Nikouei, 2020). Their ability to encapsulate diverse therapeutic agents, including hydrophobic drugs, makes them ideal for OSCC due to inherent biocompatibility.

The versatility of LNPs, including solid lipid nanoparticles (SLNs) and nanostructured lipid carriers (NLCs), lies in their capacity to improve drug stability, control release, and enhance tissue targeting. Surface functionalization enables active tumor cell targeting, minimizing off-target effects. Furthermore, the biocompatibility and biodegradability of lipid-based formulations provide an advantageous safety profile crucial for clinical application (Nasirizadeh and Malaekeh-Nikouei, 2020; Van Der Meel et al., 2020).

#### 3.3.1 Types of LNPs and their applications

##### 3.3.1.1 Solid lipid nanoparticles (SLNs)

SLNs, comprising a solid lipid matrix, effectively carry hydrophilic and lipophilic drugs due to their controlled release and enhanced stability. However, their crystalline structure limits drug-loading capacity (Nasirizadeh and Malaekeh-Nikouei, 2020) and polymorphic transitions can affect release and stability. SLNs enhance the solubility and therapeutic index of hydrophobic drugs like DOX; Zheng G. et al. (2019) showed DOX-loaded SLNs improved tumor accumulation and reduced systemic toxicity (Zheng G. et al., 2019). SLN efficiency depends on lipid composition and crystallinity, with partially crystalline lipids offering more controlled release (Olbrich et al., 2002; Wissing and Müller, 2002). Studies have investigated SLNs for the delivery of chemotherapeutics like DOX and paclitaxel to OSCC, leveraging their capacity to encapsulate hydrophobic drugs and potentially improve their accumulation and safety profile. To overcome the limitations of conventional SLNs, hybrid systems incorporating polymeric stabilizers or novel lipids like glyceryl behenate have been explored. Jannin et al. (2018) showed that glyceryl behenate improves encapsulation efficiency and drug release (Jannin et al., 2018). Further research is needed for SLNs in combinatorial therapies, co-delivering agents with different solubility profiles. Jain et al. (2023) demonstrated enhanced bioavailability and synergistic effects in cancer models using co-loaded SLNs (Mirchandani et al., 2021; German-Cortés et al., 2023; Jain et al., 2023). Despite their promise, challenges like stability, lipid-protein interactions, and variable drug release persist. Emerging strategies, such as folate-functionalized SLNs, show potential for improved tumor specificity and reduced off-target effects (Khatri et al., 2020), representing ongoing optimization efforts for clinical application.

##### 3.3.1.2 Nanostructured lipid carriers (NLCs)

An advancement of SLNs, integrate solid and liquid lipids into their matrix, offering higher drug-loading efficiency and greater flexibility in release kinetics by reducing matrix crystallinity (Nasirizadeh and Malaekeh-Nikouei, 2020). NLCs offer potential advantages over SLNs for OSCC DDS due to their higher drug loading capacity and flexibility in releasing various therapeutic agents relevant to oral cancer treatment. Despite their potential, scalability remains a major challenge; ensuring uniform particle size, encapsulation efficiency, and batch-to-batch reproducibility is critical for regulatory approval (Nasirizadeh and Malaekeh-Nikouei, 2020). Developing continuous manufacturing processes, such as microfluidic-based techniques, may offer precision in nanoparticle formulation. Furthermore, NLC flexibility could be leveraged to co-deliver RNA-based therapeutics alongside chemotherapeutic agents, a promising yet underexplored avenue in oral cancer therapy (Nasirizadeh and Malaekeh-Nikouei, 2020; Xu et al., 2020).

#### 3.3.2 EPR effect

The EPR effect enables passive targeting of nanoparticles to tumor tissues due to their leaky vasculature. SLNs and NLCs leverage this to localize chemotherapeutic agents within tumors, reducing systemic toxicity. However, tumor heterogeneity, characterized by irregular vascularization and variable interstitial pressure, significantly challenges the EPR effect’s consistency in OSCC. Studies comparing nanoparticle penetration in 2D versus 3D tumor models highlight current *in vitro* platforms’ limitations in predicting *in vivo* efficacy (Goodarzi et al., 2021). Dense ECMs in OSCC, for instance, limit nanoparticle diffusion, underscoring the need for ECM-modifying strategies. Proposed approaches include co-delivery of ECM-degrading enzymes, such as collagenase, to improve nanoparticle penetration (Yao et al., 2020). Additionally, emerging technologies like ultrasound-mediated nanoparticle delivery may transiently enhance vascular permeability, augmenting the EPR effect (Ingram et al., 2020). LNPs can leverage the EPR effect for passive accumulation in OSCC tumors, but the dense ECM requires strategies to enhance their penetration for effective drug delivery throughout the tumor.

#### 3.3.3 Biocompatibility and biodegradability

The biocompatibility and biodegradability of lipid-based nanoparticles are critical for clinical acceptance. Some studies indicate that biodegradable lipid matrices can minimize cytotoxicity, a key concern in oral cancer therapies (Syama et al., 2022; Amiri, Ziaei Chamgordani and Ghourchian, 2024). However, the safety of degradation products, particularly from modified or synthetic lipids, remains insufficiently studied. Long-term studies are needed to assess the immunological and metabolic impacts of lipid degradation products, especially in preclinical models simulating chronic drug administration, given their potential effect on treatment outcomes in patients with compromised metabolic functions (Di et al., 2023; Preeti et al., 2023; Back et al., 2024). For LNP-based DDS in OSCC, ensuring biocompatibility and understanding the fate and potential toxicity of lipid degradation products is essential for patient safety, particularly with local or repeat administration. Additionally, integrating advanced computational models with experimental approaches can predict nanoparticle degradation behavior under physiological conditions (DeLoid et al., 2015; Ramezanpour et al., 2016; Rezić and Somogyi Škoc, 2024).

#### 3.3.4 Active targeting capabilities

Active targeting of LNPs involves functionalizing their surfaces with ligands (e.g., folate, peptides, antibodies) that bind to overexpressed receptors in cancer cells (Van Der Meel et al., 2020). For instance, Bhattacharya et al. demonstrated that surface-functionalized LNPs can selectively target oral cancer cells, improving therapeutic efficacy (Bhattacharya, 2022). Similar to polymeric and inorganic nanoparticles, LNPs can be functionalized with ligands or antibodies targeting relevant OSCC receptors like CD44 or EGFR to achieve active targeting and improve therapeutic efficacy. However, receptor downregulation after repeated targeting remains a significant challenge. To address this, developing multifunctional LNPs capable of targeting multiple pathways or receptors simultaneously could improve therapeutic robustness. Biomimetic approaches, such as coating LNPs with cancer cell-derived membranes, may also enhance targeting specificity and reduce receptor downregulation (Gong et al., 2020).

#### 3.3.5 Controlled drug release

Stimuli-responsive LNPs that release their payloads in response to environmental triggers—such as pH, temperature, or enzymatic activity—have shown promise in preclinical studies (Hirai et al., 2021). For example, SLNs encapsulating paclitaxel exhibited enhanced cytotoxicity and reduced systemic toxicity by preferentially releasing the drug in acidic tumor microenvironments (Khatri et al., 2020). Stimuli-responsive LNPs, particularly those sensitive to the acidic OSCC TME, are being explored to achieve controlled and localized drug release, enhancing efficacy and minimizing systemic side effects. To further improve these systems, integrating real-time monitoring capabilities, such as fluorescent or MRI-active probes, into LNP designs could enable clinicians to dynamically adjust treatment protocols, aligning drug release with the evolving tumor microenvironment and maximizing therapeutic efficacy (Sun et al., 2021).

#### 3.3.6 Stability in the bloodstream

LNPs face challenges related to aggregation and degradation in systemic circulation, limiting therapeutic efficacy. Coating strategies like PEGylation effectively enhance nanoparticle stability and prolong circulation time (Tian et al., 2022). However, PEGylation’s immunogenicity, particularly the “accelerated blood clearance” phenomenon, remains a concern. Recent studies explore alternative coatings, such as zwitterionic polymers and polysaccharides, offering similar stability benefits without immunogenicity issues (Peng et al., 2020). Personalized nanoparticle formulations, tailored to individual patient immune profiles, could further enhance LNP clinical applicability. Maintaining LNP stability in systemic circulation is crucial for successful delivery to OSCC tumors, especially for metastatic disease, and strategies like PEGylation or alternative coatings are employed to prolong their bloodstream lifespan (Chaudhary et al., 2024).

#### 3.3.7 Efficient tumor penetration

Efficient penetration of LNPs into oral cancer tissues is critical but challenging due to the dense ECM and tumor heterogeneity. Studies show NLCs loaded with compounds like curcumin can enhance nanoparticle diffusion in tumor spheroids, indicating potential to overcome ECM barriers (Xu et al., 2020). However, the high collagen content and dense fibrotic structure of oral cancer ECM restrict nanoparticle infiltration (Yao et al., 2020). A promising strategy to address this involves co-delivery of ECM-modulating agents, such as collagenase, which selectively degrades the matrix and enhances nanoparticle penetration without harming healthy tissue (Netti et al., 2000).

The TME also significantly influences nanoparticle interaction, with variations in pH, hypoxia, and receptor expression affecting efficiency (Sun et al., 2021). Stimuli-responsive LNPs, which release drugs in response to acidic or hypoxic conditions, are emerging as a promising approach to enhance specificity and minimize off-target effects (Zhu et al., 2019). Tumor heterogeneity further complicates consistent nanoparticle penetration. Recent studies highlight the importance of advanced imaging and computational modeling to predict LNP behavior *in vivo* and guide the development of more effective, personalized delivery systems (Di et al., 2023). Overcoming the dense OSCC ECM is a major barrier; strategies involving ECM degradation or stimuli-responsiveness based on the TME are being explored to improve LNP distribution within oral tumors. These innovative approaches enable tailoring LNP delivery to overcome complex TME barriers, offering improved oral cancer treatment outcomes.

#### 3.3.8 Limitations, toxicity concerns and future directions

Although LNPs are generally considered safe, their accumulation in vital organs and the long-term effects of degradation products necessitate thorough investigation (Yang L. et al., 2022). While largely biocompatible, prolonged safety and potential accumulation of LNPs or their degradation products in organs susceptible to systemic exposure in OSCC treatment demand further scrutiny. High-resolution imaging coupled with multi-omics approaches could offer valuable insights into nanoparticle biodistribution and toxicity profiles during extended treatment courses (Di et al., 2023).

##### 3.3.8.1 Manufacturing challenges

The scalability of LNP production remains a significant hurdle for clinical implementation. Ensuring reproducibility in particle size, drug encapsulation, and release profiles during industrial production is crucial. Advances in manufacturing technologies, such as microfluidic systems, will play a pivotal role in overcoming these challenges and facilitating widespread LNP use (Roces et al., 2020). Scaling up LNP production for OSCC DDS to meet clinical demand presents manufacturing challenges related to reproducibility and quality control, requiring advancements in production technologies.

LNPs represent a groundbreaking advancement in oral cancer therapy, offering solutions to critical issues like poor drug bioavailability, systemic toxicity, and lack of targeting specificity (Syama et al., 2022). Despite their promise, addressing challenges like long-term toxicity, tumor heterogeneity, and production scalability requires fostering interdisciplinary research and technological advancements.

### 3.4 Stimuli-responsive DDS based on nanotechnology for oral cancer treatment

Stimuli-responsive DDS have revolutionized nanotechnology, offering transformative potential for oral cancer treatment by leveraging the TME unique physiological and biochemical characteristics for precise, controlled, and localized drug delivery (Zhu et al., 2019). Unlike conventional approaches limited by systemic toxicity and poor tumor selectivity, stimuli-responsive DDS respond to specific TME triggers, providing spatiotemporal control over drug release and tailoring treatment to the dynamic tumor environment (Li et al., 2019).

**FIGURE 2:**
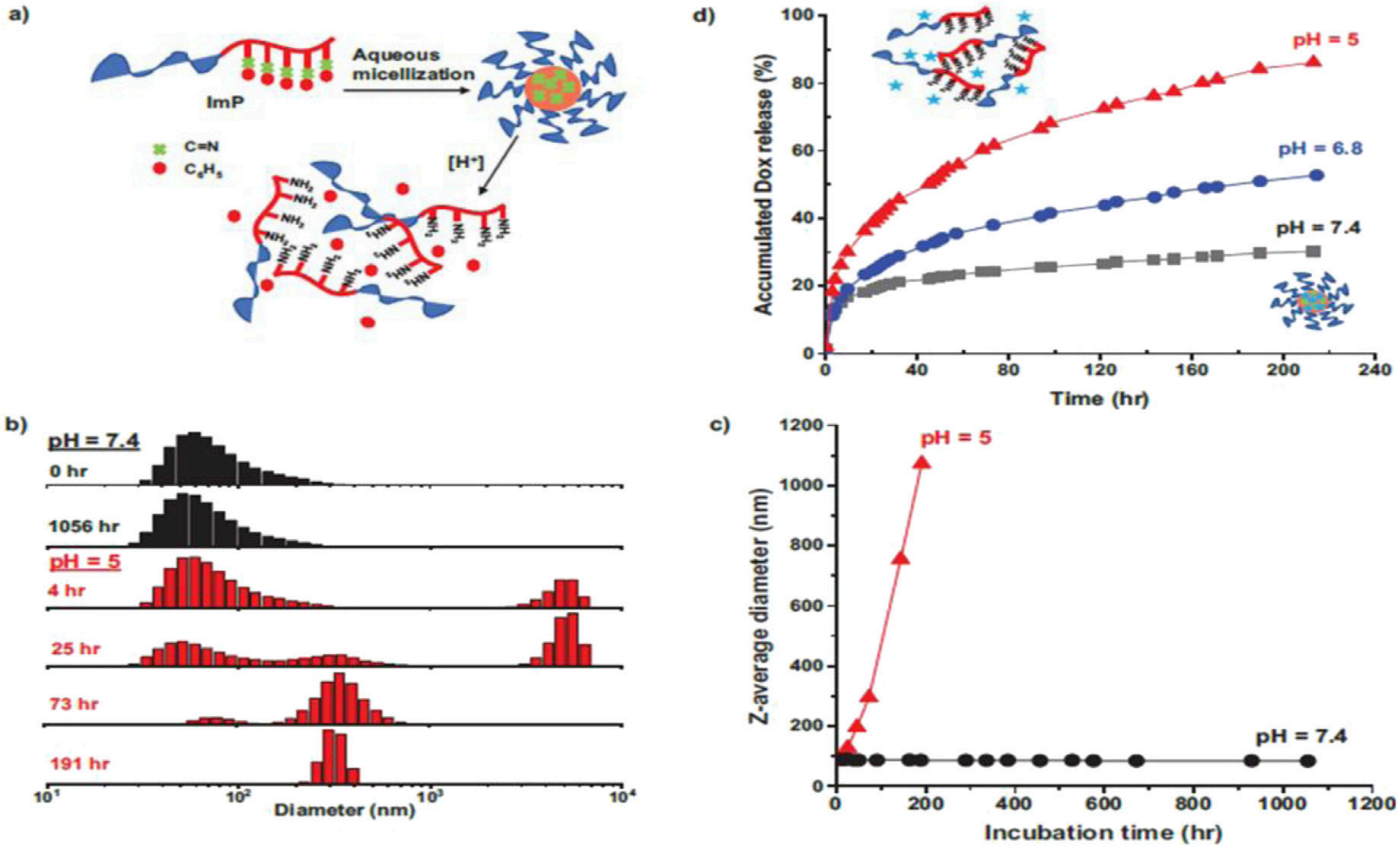
**(a)** Schematic illustration of acid-responsive dissociation of self-assembled micelles; **(b)** overlaid DLS diagrams and **(c)** evolution of Z-average diameter of ImP micelles at 1 mg mL^−1^, incubated at physiological pH = 7.4 and acidic pH = 5.0; and **(d)** %Dox release over incubation time at acidic. pHs = 5.0 and 6.8, compared with physiological pH = 7.4 (Hu and Oh, 2020).

Integrating stimuli-responsive elements—such as pH, temperature, and reactive oxygen species (ROS)—into DDS represents a promising frontier in cancer therapy. These systems optimize anticancer agent efficacy while minimizing off-target effects, thereby enhancing the therapeutic index, Figure 2 (Hu and Oh, 2020). The incorporation of targeting ligands and multimodal mechanisms further expands their versatility and precision, making them integral to future cancer treatment strategies (Liang et al., 2021). For OSCC, stimuli-responsive nanoparticles can exploit tumor-specific triggers like acidic pH, elevated ROS levels, or specific enzymatic activity within the TME to achieve targeted and controlled drug release, minimizing systemic exposure and maximizing local efficacy.

#### 3.4.1 pH-sensitive DDS: harnessing tumor acidity

The acidic of the TME, driven by the Warburg effect and resulting lactate accumulation, provides a natural trigger for pH-sensitive drug delivery systems (DDS). This allows selective therapeutic agent release at the tumor site, maximizing local drug concentrations while minimizing systemic exposure (Chen H. et al., 2020). The acidic pH (approximately 6.5–6.8) in the OSCC TME makes pH-sensitive nanoparticles highly relevant for targeted drug release in oral cancer.

An example is thermo-pH dual-responsive polymersomes loaded with a poly (N-isopropylacrylamide)-doxorubicin (PNIPAM-DOX) conjugate. These PEG-PTMBPEC polymersomes are stable physiologically but disassemble at acidic tumor pH (<6.5) due to acetal bond degradation. Concurrently, the thermoresponsive hydrogel forms a gel at body temperature, sustaining drug release. This system enhances DOX bioavailability, prolongs circulation, and significantly inhibits tumor growth *in vivo*, with reduced systemic toxicity compared to free DOX (Oroojalian et al., 2018).

However, tumor heterogeneity, specifically variations in pH across tumor regions and intracellular compartments, poses challenges to consistent pH-sensitive DDS performance, leading to unpredictable drug release. To overcome this, hybrid systems combining pH sensitivity with other triggers like temperature or ROS are being explored to improve specificity and therapeutic consistency (Li et al., 2020). pH-Sensitive polymeric nanoparticles and polymersomes are being investigated for targeted chemotherapeutic delivery to the acidic OSCC tumor site, showing improved efficacy in preclinical models.

#### 3.4.2 Temperature-sensitive DDS: exploiting local hyperthermia

Temperature-sensitive DDS exploit TME fluctuations, especially those induced by hyperthermia, to control drug release. Elevated tumor temperatures (42°C–45°C), driven by inflammation, metabolic activity, or external heat, trigger phase transitions in thermosensitive materials. These systems, often hydrogels or nanoparticles, remain liquid at physiological temperatures but undergo a sol-gel transition at elevated temperatures, enabling localized and sustained drug release (Li et al., 2012). Temperature-sensitive nanoparticles can be combined with hyperthermia (e.g., induced by PTT) to trigger localized drug release within OSCC tumors, offering synergistic therapeutic effects.

When combined with hyperthermia-based therapies, temperature-sensitive DDS demonstrate synergistic effects. For instance, thermosensitive hydrogels loaded with DOX and gold nanoparticles have shown enhanced tumor ablation (Li et al., 2016). The photothermal properties of gold nanoparticles amplify localized heating, facilitating precise drug release and improved therapeutic efficacy. Despite their promise, challenges remain in achieving uniform tumor heating and precise phase transition control. Hybrid systems integrating temperature sensitivity with pH or ROS responsiveness are being developed to address these issues, enabling broader clinical applicability (Zhang et al., 2021).

#### 3.4.3 ROS-sensitive DDS: leveraging oxidative stress

Elevated ROS levels in the TME, driven by hypoxia and rapid cell division, present a promising trigger for DDS. ROS-sensitive systems are designed with linkages, such as thioketal bonds, that undergo oxidative cleavage in response to ROS, ensuring selective drug release at the tumor site (Shi et al., 2018). In OSCC models, ROS-sensitive nanoparticles show great potential. For example, curcumin-loaded nanoparticles release their therapeutic payload in response to elevated ROS levels, enhancing localized drug delivery and reducing systemic toxicity (Zhang Z. et al., 2020). The elevated ROS levels in the hypoxic regions of OSCC tumors provide a relevant stimulus for ROS-sensitive nanoparticles, enabling localized drug release and enhancing therapeutic effects, as demonstrated with systems like curcumin-loaded nanoparticles. However, variability in ROS levels across tumor regions and patients remains a significant challenge. To optimize drug delivery and improve therapeutic precision, real-time imaging of ROS levels integrated with DDS is being explored (Zhang et al., 2021).

### 3.5 Emerging use of exosomes as natural drug delivery vehicles

Exosomes, nanosized vesicles (30–150 nm), have gained significant attention as promising natural vehicles for targeted drug delivery in oral cancer therapy. These endogenous vesicles offer unique advantages, including biocompatibility, intrinsic targeting capabilities, and the ability to traverse biological barriers, setting them apart from synthetic drug delivery systems (Familtseva et al., 2019). Their natural origin leverages cellular machinery, providing a distinct edge in overcoming conventional drug delivery limitations like systemic toxicity and low target specificity. Given their endogenous origin and biological properties, exosomes are being explored as a novel approach for targeted drug delivery in OSCC, potentially offering advantages in biocompatibility and inherent targeting compared to synthetic nanoparticles (Bozyk et al., 2023).

#### 3.5.1 Biocompatibility and immunological inertness

A key advantage of exosomes is their exceptional biocompatibility, primarily due to their lipid bilayer structure resembling parent cells. This minimizes immune recognition and inflammatory responses common with synthetic nanoparticles, making exosomes particularly suitable for clinical applications and crucial for reducing systemic toxicity in cancer treatments (Wu et al., 2024). Studies demonstrate that exosomes from various cellular sources exhibit low immunogenicity and prolonged circulation times, enhancing encapsulated drug bioavailability by evading rapid clearance (Bagheri et al., 2020). Furthermore, certain exosomal surface proteins, like CD47, mediate immune evasion, though precise mechanisms remain under investigation (Cheng et al., 2021). Despite these advantages, challenges persist in fully understanding exosome biodistribution and immune system interactions, warranting further research. The high biocompatibility and low immunogenicity of exosomes are particularly attractive for OSCC DDS, potentially leading to better tolerability compared to some synthetic nanocarriers.

#### 3.5.2 Exosome-mediated targeting in oral cancer therapy

Exosomes are inherently equipped with targeting properties conferred by their surface composition, reflecting their parent cells’ proteins, lipids, and nucleic acids. This composition facilitates selective binding to target cells via interactions with overexpressed receptors such as EGFR, integrins, and folate receptors, commonly found on oral cancer cells (Nguyen Cao et al., 2021). These targeting mechanisms reduce off-target effects, significantly mitigating collateral damage from conventional chemotherapy. Exosomes possess intrinsic targeting capabilities towards OSCC cells due to specific surface proteins and lipids that interact with receptors overexpressed in oral cancer, enabling more precise drug delivery (Ma et al., 2024).

#### 3.5.3 Exosomes for RNA delivery: small interfering RNA (siRNA) and microRNA (miRNA) in oral cancer

Exosomes are emerging as promising carriers for RNA-based therapies in oral cancer, particularly for delivering siRNA and miRNA. They offer a protective environment that shields RNA molecules from enzymatic degradation in the bloodstream and improves cellular uptake (Wang K. et al., 2021), enabling targeted modulation of tumor-associated genes. The delivery of miRNA mimics such as let-7 has shown potential to suppress oncogenic pathways and metastasis (Otmani and Lewalle, 2021). Despite promising results, challenges persist in optimizing RNA loading, stability, and release kinetics within exosomes to achieve consistent therapeutic outcomes. Ongoing research in RNA engineering and loading methods is essential to fully harness their potential. Exosomes offer a compelling platform for delivering siRNA and miRNA to OSCC cells, protecting RNA from degradation and enabling targeted gene silencing to suppress oncogenic pathways (Sayyed et al., 2021).

#### 3.5.4 Challenges in exosome-based drug delivery

While exosomes show considerable promise, several challenges hinder their clinical implementation. A primary limitation is the difficulty in isolating and purifying exosomes at scale, as current techniques (ultracentrifugation, size-exclusion chromatography, immunoaffinity capture) yield heterogeneous populations, complicating standardization (Lee et al., 2020). Advances in isolation technologies are urgently needed to ensure homogeneity and reproducibility. Loading therapeutic agents, especially large or hydrophilic drugs, into exosomes presents another challenge; techniques like electroporation, sonication, and chemical conjugation may compromise exosome integrity and functionality (Nguyen Cao et al., 2021). Additionally, although generally considered immunologically inert, exosomes’ potential to trigger immune responses, particularly in patients with underlying immune disorders, cannot be entirely excluded. Strategies to reduce immunogenicity, such as using autologous exosomes or surface modifications, are under active investigation. Challenges in the clinical translation of exosome-based DDS for OSCC include scalable isolation, efficient drug/RNA loading, and ensuring consistent targeting and minimal immunogenicity.

#### 3.5.5 Future perspectives on exosome-based therapies

Exosomes represent a transformative and versatile platform for drug delivery in oral cancer therapy, offering advantages like biocompatibility, intrinsic targeting, and the ability to deliver complex therapeutics such as RNA. These features position exosomes as superior alternatives to synthetic delivery systems. However, addressing challenges related to scalability, standardization, and cargo loading is essential for clinical translation (Nguyen Cao et al., 2021). The future of exosome-based therapies lies in integrating these natural carriers with advances in nanotechnology and molecular biology to optimize their design and functionality. Innovations such as engineered exosomes with enhanced targeting specificity, improved drug loading capacity, and real-time monitoring capabilities could significantly enhance their therapeutic potential. As interdisciplinary research continues to refine these systems, exosomes hold the promise of revolutionizing oral cancer treatment and advancing personalized medicine (Familtseva et al., 2019; Wu et al., 2024).

## 4 Localized DDS

Localized DDS offer precise therapeutic targeting for oral tumors and OPMLs, addressing challenges such as systemic toxicity, drug resistance, and the oral cavity’s unique anatomy. Advances in materials science, particularly in intelligent hydrogels, mucoadhesive patches, and nanofiber matrices, have significantly expanded the possibilities for localized drug release, improving clinical outcomes and minimizing side effects.

### 4.1 Intelligent hydrogels for localized drug delivery in oral tumors

**FIGURE 3:**
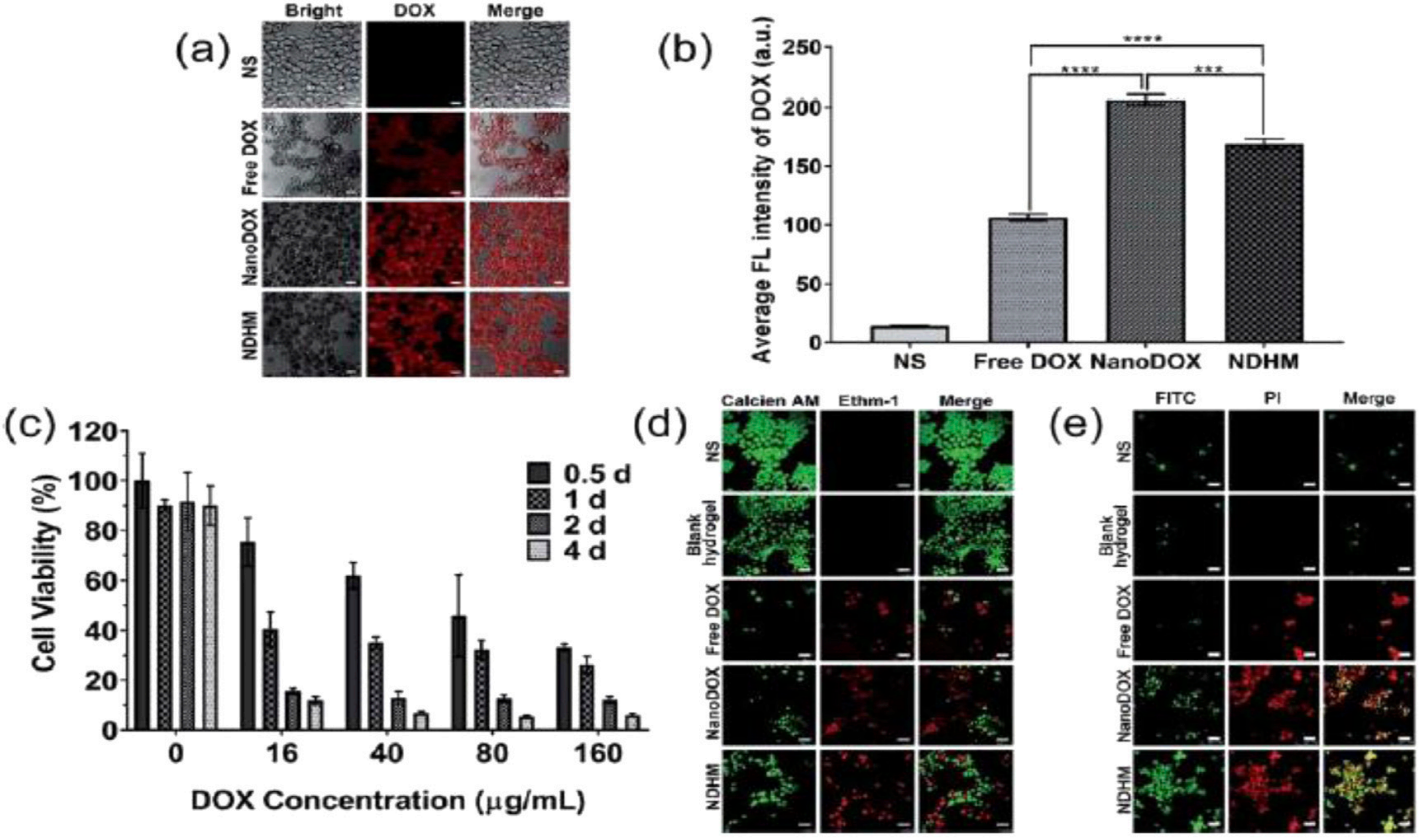
**(a)** CLSM images showing the cellular distribution of DOX (red) in SCC-15 cells that were treated with NS, free DOX, NanoDOX, and NDHM at a 16 mg mL^−1^ DOX concentration in the medium (scale bar = 25 mm); **(b)** the fluorescence quantitative analysis of the cellular uptake of DOX. Data represent means _ SD (n = 3), ***P < 0.001, ****P < 0.0001. **(c)** Relative cell viabilities of SCC-15 cells after treatments with different concentrations of DOX in HA-MMP hydrogels for 0.5 days, 1 day, 2 days, and 4 days. Data represent means _ SD (n = 3). **(d)** CLSM images of SCC-15 cells stained with calcein AM and Ethm-1 upon different treatments with NS, blank hydrogel, free DOX, NanoDOX, and NDHM at a 16 mg mL^−1^ DOX concentration in the medium (scale bar = 50 mm). **(e)** CLSM images of SCC-15 cells stained with FITC and PI upon different treatments with NS, blank hydrogel, free DOX, NanoDOX or NDHM at a 16 mg mL^−1^ DOX concentration in the release medium (scale bar = 50 mm) (Li et al., 2019).

Intelligent hydrogels are three-dimensional polymeric networks designed to respond to specific environmental stimuli like temperature, pH changes, or enzymatic activity. This responsiveness enables DDS capable of controlled therapeutic agent release at the oral tumor target site (Zhao et al., 2022). Beyond drug delivery, intelligent hydrogels represent a convergence of material science, biology, and oncology, opening new possibilities for personalized and effective treatment. Ongoing efforts aim to refine hydrogel formulations not only for optimized drug release but also for tailoring them to specific tumor microenvironment conditions, improving treatment outcomes, Figure 3 (Li et al., 2019). The versatility of these hydrogels, in adapting to different stimuli, further enhances their potential in targeted cancer therapy.

#### 4.1.1 Advantages of intelligent hydrogels

Intelligent hydrogels offer significant advantages in cancer treatment, particularly for localized oral cavity therapies. Their biocompatibility and biodegradability minimize adverse reactions, making them ideal for use with toxic anticancer drugs while enhancing patient tolerance, crucial for compliance and quality of life (Remiro et al., 2022).

A major benefit is their injectable liquid state and subsequent *in situ* gelation at the tumor site. This minimally invasive approach is especially advantageous in the sensitive oral cavity, were surgical interventions risk complications. Additionally, hydrogels provide controlled drug release, ensuring sustained therapeutic concentrations over extended periods, essential for managing chronic conditions like oral cancer requiring long-term treatment efficacy (Zhao et al., 2022).

Another key advantage is their capacity for simultaneous multi-drug delivery, enabling combination therapies with synergistic outcomes. This multidrug strategy is valuable in overcoming drug resistance by targeting multiple pathways. Studies demonstrate hydrogels’ potential in improving therapeutic indices, reducing systemic exposure, and addressing complex tumor microenvironment dynamics, positioning them as a transformative tool in localized cancer therapy (Li et al., 2016).

#### 4.1.2 Stimulus-responsive hydrogels for enhanced treatment specificity

Thermosensitive hydrogels undergo a sol-to-gel transition with temperature changes. Injectable as a liquid at room temperature, they solidify at body temperature, forming a depot for sustained drug release (Li et al., 2016). Incorporating nanoparticles, like carboplatin, can further improve therapeutic agent sustained release, showing promise where precise drug delivery is crucial (Thakur et al., 2020).

##### 4.1.2.1 pH-sensitive hydrogels

Tumor microenvironments often exhibit acidic pH due to increased glycolytic activity, making pH-sensitive hydrogels effective for tumor targeting. These hydrogels are designed to preferentially release their drug payload in acidic conditions, enhancing treatment specificity and minimizing healthy tissue exposure. For instance, pH-sensitive self-assembling peptide hydrogels have been developed to optimize drug release in response to the acidic conditions characteristic of oral cancer tissues (Zhao et al., 2022).

#### 4.1.3 Specific applications of intelligent hydrogels in oral tumor treatment

##### 4.1.3.1 Thermosensitive hydrogel loaded with gambogic acid (GA)

This formulation provides sustained GA release at the tumor site, exhibiting significant antitumor effects and modulating the tumor immune microenvironment to enhance systemic antitumor responses (Zhang D. et al., 2020).

##### 4.1.3.2 Thermosensitive hydrogel loaded with suberoylanilide hydroxamic acid (SAHA) and cisplatin (DDP)

This combination synergistically enhances therapeutic efficacy and addresses drug resistance, a common obstacle in cancer treatment (Li et al., 2012).

##### 4.1.3.3 Photosensitive hydrogel with ink nanoparticles and dihydroartemisinin (DHA)

This innovative hydrogel integrates photothermal therapy (PTT) with chemotherapy. Ink nanoparticles, upon light irradiation, generate localized heat, promoting DHA release for a dual therapeutic effect. This approach is promising for accessible tumors where light exposure can be controlled (Chen D. et al., 2020).

##### 4.1.3.4 Enzyme-sensitive hydrogel loaded with DOX

Designed to respond to matrix metalloproteinases (MMPs) overexpressed in the oral cancer microenvironment, this hydrogel enables targeted DOX release precisely at the tumor site, enhancing targeting accuracy by exploiting oral cancer’s biochemical markers (Liang et al., 2021).

Intelligent hydrogels mark a significant advance in localized drug delivery for oral tumors. Their stimulus-responsive nature within the tumor microenvironment ensures controlled, targeted drug release, enhancing efficacy and minimizing systemic side effects (Zhao et al., 2022). As research progresses, these hydrogels are poised to play a critical role in oncology, with future studies focusing on optimizing formulations, improving responsiveness, and validating clinical effectiveness. The intersection of hydrogel technology and cancer therapy offers an exciting opportunity to redefine treatment paradigms through more precise and personalized approaches.

### 4.2 Mucoadhesive patches for OPMLs

Mucoadhesive systems significantly advance localized drug delivery in the oral cavity, particularly for Oral Potentially Malignant Lesions (OPMLs). These systems adhere firmly to mucosal surfaces, ensuring prolonged drug retention at the application site, crucial for overcoming rapid salivary clearance and mucosal turnover (Holpuch et al., 2012; Elbayoumi et al., 2015). For OSCC and OPMLs, mucoadhesive patches are particularly advantageous for direct lesion delivery, counteracting salivary flow and tissue movement.

Mucoadhesion relies on physicochemical interactions between adhesive polymers and the mucus layer, ensuring consistent drug presence for sustained delivery (Elbayoumi et al., 2015). These systems also maintain optimal drug concentration despite oral cavity fluctuations (Mabrouk et al., 2023). Common polymers like poly (acrylic acid), chitosan, and cellulose derivatives exhibit strong bioadhesive properties through hydrogen bonding, electrostatic interactions, and Van der Waals forces (Cui et al., 2019). These materials enhance adhesion and enable controlled drug release, reducing reapplication frequency and maintaining therapeutic levels, critical for treating dysplastic lesions while avoiding additional mucosal damage (Elbayoumi et al., 2015).

Mucoadhesive systems optimize pharmacokinetics and pharmacodynamics by improving local bioavailability and reducing systemic exposure, ensuring precise drug localization and mitigating side effects. A notable application is the localized delivery of chemopreventive agents, such as fenretinide, a synthetic retinoid effective in managing OPMLs. Fenretinide induces terminal differentiation and apoptosis in dysplastic keratinocytes, sparing normal cells (Holpuch et al., 2012; Wang et al., 2022). Preclinical studies show fenretinide-loaded mucoadhesive patches achieve therapeutic concentrations, inhibiting dysplastic cell proliferation and promoting differentiation markers (Desai et al., 2011). Fenretinide’s mechanism, mediated by retinoic acid receptors (RARs) and retinoid X receptors (RXRs), supports its targeted local efficacy, mitigating dose-dependent systemic toxicities (Han et al., 2015; Ishikawa et al., 2022).

Incorporating agents like fenretinide into mucoadhesive patches enhances local bioavailability, sustains drug presence, and minimizes systemic exposure. This dual benefit is crucial for effective OPML chemoprevention and treatment, offering a non-invasive, patient-friendly alternative that may reduce recurrence and progression to OSCC. Overall, mucoadhesive patches provide a versatile platform for oral cancer prevention, adaptable for various active compounds.

#### 4.2.1 Advantages and clinical relevance of mucoadhesive patches

Mucoadhesive patches offer distinct advantages as a non-invasive, localized drug delivery method, particularly in the challenging oral cavity.

##### 4.2.1.1 Non-invasive and patient-friendly administration

These patches provide a comfortable and straightforward application, enhancing patient compliance and enabling at-home treatment, which is especially beneficial for managing chronic or recurrent conditions like OPMLs (Liu Xiaoqin et al., 2022).

##### 4.2.1.2 Enhanced local bioavailability and therapeutic efficiency

By maintaining prolonged drug contact with the lesion, mucoadhesive patches ensure higher local drug concentrations, optimizing therapeutic efficacy. This localized delivery reduces dosage and potential adverse effects, crucial for managing dysplastic lesions where precise, minimally invasive interventions are needed to protect delicate mucosal tissue (Elbayoumi et al., 2015; Liu Xiaoqin et al., 2022).

##### 4.2.1.3 Reduced systemic side effects

Targeted drug delivery via mucoadhesive patches minimizes systemic exposure, offering a safer alternative to systemic administration. This is particularly advantageous for OPMLs, where systemic distribution of cytotoxic or differentiation-inducing agents might cause undesirable side effects. By confining drug action to the lesion site, these patches improve overall treatment safety and tolerability. Reducing systemic toxicity is a major advantage for OSCC, allowing potentially higher local doses with fewer side effects compared to systemic chemotherapy (Liu Xiaoqin et al., 2022; Cheng et al., 2023).

Mucoadhesive systems, especially biodegradable patches, represent a transformative approach in the localized treatment of OPMLs. Their ability to enhance drug retention, improve therapeutic efficiency, and mitigate systemic side effects underscores their clinical utility (Liu Xiaoqin et al., 2022). As research progresses, their scope is likely to expand beyond oral lesions, with ongoing efforts focused on optimizing formulations and evaluating efficacy across diverse clinical conditions.

### 4.3 Nanofibers and three-dimensional matrices in the treatment of advanced oral lesions

Nanofibers have emerged as a transformative tool in localized drug delivery, particularly for advanced oral lesions. These nanoscale structures, fabricated from biocompatible and biodegradable polymers, offer enhanced flexibility and adaptability for therapeutic applications. They provide a versatile platform for targeted, controlled, and multi-drug release, addressing key challenges posed by the complex pathophysiology of these conditions (Liu Y. et al., 2022; Kim et al., 2023).

#### 4.3.1 Advantages of nanofibers as drug delivery systems

Nanofiber-based DDS present several advantages for advanced oral lesions:

##### 4.3.1.1 High drug-loading capacity

Their porous structure and high surface area-to-volume ratio allow substantial drug loading, crucial for multi-drug therapies targeting heterogeneous tumor cells and overcoming resistance (Bishnoi et al., 2023).

##### 4.3.1.2 Controlled release profile

Drug release can be finely tuned by modifying composition, diameter, and porosity, ensuring sustained therapeutic concentrations at the target site, reducing re-administration frequency, and mitigating side effects from fluctuating drug levels (Bishnoi et al., 2023).

##### 4.3.1.3 Biocompatibility and biodegradability

Composed of biocompatible polymers, nanofibers minimize adverse reactions and gradually degrade within tissue, crucial for long-term treatment where foreign materials could trigger inflammation (Subramanian et al., 2013).

##### 4.3.1.4 Adaptability to oral anatomy

Nanofibers can be engineered into membranes or 3D matrices conforming to the oral cavity’s dynamic environment, ensuring effective localized delivery in challenging anatomical settings (Kim et al., 2023).

#### 4.3.2 Multi-component nanofiber systems

A notable application involves polyvinyl alcohol (PVA)-based membranes loaded with glucose oxidase (GOx), manganese dioxide (MnO_2_), and rapamycin, exemplifying a multi-modal therapeutic approach:

##### 4.3.2.1 Rapamycin

A chemotherapeutic agent inhibiting the mTOR pathway, a key driver of oral cancer cell proliferation. Its localized delivery enhances therapeutic effects while minimizing systemic toxicity (Kim et al., 2023).

##### 4.3.2.2 Gox

Catalyzes glucose breakdown, exploiting the Warburg effect to deplete cancer cell energy and produce hydrogen peroxide (H_2_O_2_), inducing oxidative stress (Kim et al., 2023).

##### 4.3.2.3 MnO_2_

Enhances oxidative stress by reducing intracellular glutathione (GSH) levels, sensitizing cancer cells to ROS. MnO_2_ also mitigates hypoxia and catalyzes a Fenton-like reaction, producing hydroxyl radicals (•OH) that promote apoptosis (Kim et al., 2023).

This combination demonstrates the synergistic potential of multi-component nanofiber systems in addressing tumor heterogeneity and enhancing therapeutic efficacy.

#### 4.3.3 Integrating nanofiber technology with PDT

Nanofibers are compatible with advanced therapeutic modalities like Photodynamic Therapy (PDT), which utilizes a photosensitizer activated by specific light wavelengths to generate ROS and induce cytotoxicity (Sun et al., 2022). A hybrid hydrogel system incorporating nanofibers demonstrates this synergy. The system combines mesoporous silica nanoparticles (MSNs) loaded with IR820, a cyanine dye with photothermal properties, and DOX, an established chemotherapeutic agent (Wu et al., 2021). Upon near-infrared (NIR) light irradiation, this system achieves synchronized photothermal and chemotherapeutic effects:

##### 4.3.3.1 IR820

Absorbs NIR light, generating thermal energy that induces localized hyperthermia and apoptosis in tumor cells, complementing DOX’s chemotherapeutic action (Wu et al., 2021).

##### 4.3.3.2 DOX

Delivered controllably from MSNs, disrupts DNA synthesis in cancer cells. Oxidative stress from IR820 activation accelerates its release through ROS-sensitive diselenide linkages, creating a precisely triggered, potent anti-cancer effect (Wu et al., 2021).

This multi-modal system exemplifies the potential of nanofiber platforms to integrate diverse therapeutic strategies, offering comprehensive treatment options for advanced oral cancers.

#### 4.3.4 Discussion and future directions

The integration of nanofiber-based systems with multi-drug and multi-modal approaches significantly advances the treatment of advanced oral lesions (Sun et al., 2022). By combining chemotherapy, photothermal, and starvation therapy, these systems address critical limitations like tumor heterogeneity and resistance (Ying et al., 2020).

Future research should optimize nanofiber physicochemical properties, including degradation rates, mechanical stability, and drug-release kinetics, to enhance functionality in the oral environment. Personalized formulations tailored to individual tumor biomarkers and local microenvironments could further improve therapeutic outcomes. Clinical trials are essential to validate efficacy and safety, ensuring transition from experimental models to practical applications.

Nanofiber-based systems, especially with complementary therapies, represent a versatile and effective platform for localized drug delivery in oral cancer treatment (Bishnoi et al., 2023). Their ability to achieve controlled, multi-drug release while minimizing systemic toxicity offers significant potential to improve patient outcomes through targeted and minimally invasive strategies.

### 4.4 Micro electro mechanical drug delivery systems (MEMS) for controlled drug delivery

MEMS represent an innovative drug delivery platform that leverages microfabrication to enable precise, miniaturized, and programmable administration (Chircov and Grumezescu, 2022). Through components like micro-reservoirs, micropumps, and microvalves, they allow pulsatile, continuous, or on-demand release profiles (Tng et al., 2012). Their compact size enables minimal invasiveness, suitable for implantation or mucosal adhesion, offering precise control over drug timing and dosage for therapies requiring specific concentration profiles or triggered delivery (Mariello et al., 2023).

A key capability for complex diseases like oral cancer is the integration of sensors for real-time monitoring and biosensing within the TME, detecting changes in pH, temperature, or specific biomarkers (Tng et al., 2012). This real-time data enables adaptive, closed-loop DDS, where sensor feedback triggers or modulates drug release via micro-actuators. For instance, detecting a threshold pH in OSCC could automatically trigger microvalve opening for pH-sensitive drug delivery, ensuring precise, effective therapeutic agent administration and minimizing systemic exposure (Tng et al., 2012; Ullah et al., 2017). This programmable, responsive approach allows for personalized, real-time therapy adapting to tumor site conditions (Chircov and Grumezescu, 2022).

MEMS devices show particular promise for oral cancer therapy due to the need for localized control and the oral cavity’s unique environment. Their potential for implantation or adhesion allows targeted delivery to primary tumors or surgical margins, potentially overcoming salivary clearance and achieving high local drug concentrations with minimal side effects (Tng et al., 2012). While research explores MEMS for chemotherapy release in cancer models, specific OSCC applications are an active development area (Villarruel Mendoza et al., 2020). Integrating MEMS with nanotechnology, like nanoparticle-laden micro-reservoirs, promises enhanced drug targeting. Emerging trends include wireless-controlled MEMS and hybrid systems combining drug delivery with interventions like photothermal or PDT (Ullah et al., 2017; Kurul et al., 2025).

Challenges remain, such as ensuring long-term biocompatibility, stability in the oral environment, scalable fabrication, and safe retrieval or biodegradation. Overcoming these barriers is key to translating MEMS into effective clinical tools for OSCC.

### 4.5 Artificial intelligence (AI) in DDS for oral cancer

AI is revolutionizing the pharmaceutical and biomedical fields by optimizing drug formulations and personalizing therapies through complex data analysis (Serrano et al., 2024). Unlike traditional methods, AI algorithms precisely predict drug behavior, from pharmacokinetics to DDS performance, potentially accelerating development and reducing costs (Deori Leena Hujuri, 2024).

**FIGURE 4:**
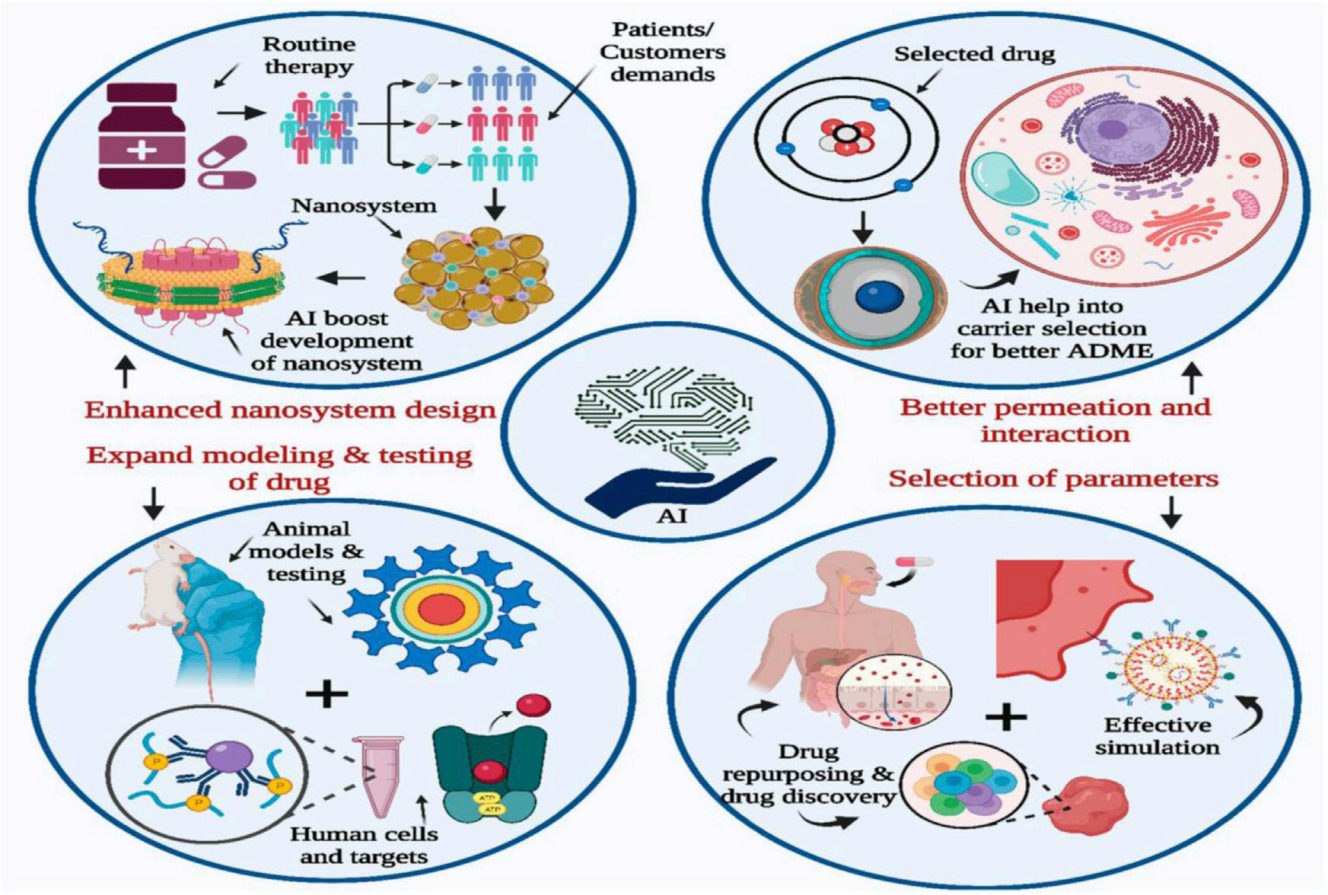
AI contribution to drug development and research. AI can be used to enhance nanosystem design, expand the present drug testing modeling system, and increase the accuracy of parameter and factor selection in drug design, drug discovery, and drug repurposing methods. It also helps to better understand the mechanism of membrane interaction with the modeled human environment by studying drug permeation, simulation, human cell targets, etc., (Vora et al., 2023).

AI’s transformative applications in drug delivery facilitate advanced DDS design via multiscale modeling and predictive algorithms. Machine learning models, for instance, optimize nanoparticle carriers by predicting encapsulation efficiency, release profiles, and biocompatibility (Figure 4) (Vora et al., 2023). These approaches hold significant potential for OSCC, specifically in overcoming the dense extracellular matrix and achieving precise targeting within the complex oral tumor microenvironment. AI models could enable nanocarrier design tailored to OSCC’s unique physiological conditions, enhancing therapeutic index (Vinay et al., 2025).

Integrating patient-specific genomic and proteomic data allows AI to guide personalized DDS design (Mirfendereski et al., 2025). Through real-time biomarker and physiological response analysis, AI can dynamically adapt drug release patterns, crucial for heterogeneous diseases like cancer (Agarwal et al., 2023). For OSCC, this could involve designing DDS responsive to a patient’s unique molecular characteristics or tumor microenvironment, optimizing efficacy while minimizing toxicity (Kapoor et al., 2024; Vinay et al., 2025).

Despite its potential, AI application in DDS for OSCC faces significant challenges: access to high-quality, OSCC-specific datasets for model training, potential algorithmic bias, and the critical need for interdisciplinary collaboration among computational scientists, pharmaceutical developers, and oral cancer oncologists (Deori Leena Hujuri, 2024; Chowdhury et al., 2025; Vinay et al., 2025). Addressing these issues is essential for realizing AI’s full potential in enhancing DDS performance and improving OSCC treatment outcomes. While current AI literature in oral cancer often focuses on diagnosis and prognosis, its role in optimizing and personalizing DDS for this complex disease is expected to grow substantially (Hassanzadeh et al., 2019; Deori Leena Hujuri, 2024; Serrano et al., 2024).

## 5 Combination therapy-based DDS

### 5.1 Advanced DDS combining chemotherapy and PDT for oral cancer treatment

While PDT and chemotherapy are established treatments for oral tongue squamous cell carcinoma (OTSCC), their integration via advanced DDS offers a synergistic approach with potential for significantly enhanced therapeutic outcomes (Shi et al., 2018; 2024). Traditional treatments like surgery, radiotherapy, and standalone chemotherapy are essential but often entail severe side effects and drug resistance (Bhattacharya, 2022). Incorporating nanotechnology into DDS enables precise, controlled therapeutic agent delivery, minimizing systemic toxicity while maximizing selective tumor cell targeting (Wang et al., 2015; Li et al., 2023).

### 5.2 Combined PTT with immunotherapy

PTT, converting near-infrared (NIR) light into localized heat for tumor ablation, shows synergistic potential when combined with immunotherapy. This combination amplifies therapeutic efficacy by directly inducing tumor cell death while enhancing immune recognition of tumor-associated antigens (TAAs) (Cheng et al., 2021).

#### 5.2.1 Biomimetic nanoparticles

Cancer cell membrane-coated nanoparticles (CCM-NPs) encapsulating photosensitizers like indocyanine green (ICG) and autophagy inhibitors such as hydroxychloroquine (HCQ) demonstrate efficient tumor targeting through homologous recognition. Poly (β-amino esters)/PLGA nanoparticles offer a stable platform for co-delivering PTT agents (e.g., ICG) and immunotherapeutic agents (e.g., HCQ) (Shi et al., 2024; Wu et al., 2024). This biomimetic approach improves cellular uptake and retention of therapeutic agents, enhancing PTT and immunotherapy efficacy. These systems also enhance drug solubility and bioavailability, enabling precise, localized delivery while minimizing systemic toxicity.

### 5.3 Advances in nanoparticle functionalization with specific ligands to enhance targeting and immune responses

Functionalizing nanoparticles with specific ligands is a key strategy to enhance the precision and therapeutic efficacy of drug delivery systems in the complex TME of OSCC (Marunganathan et al., 2024). Conjugating nanoparticles with tumor-targeting ligands, antibodies, or immunomodulatory agents enhances tumor uptake, reduces off-target effects, and can activate anti-tumor immune responses (Shi et al., 2018).

#### 5.3.1 Tumor-targeting ligands

These molecules bind selectively to overexpressed receptors or antigens on cancer cells or within the TME, promoting site-specific drug accumulation (Chehelgerdi et al., 2023). RGD peptides, for instance, target integrins (e.g., αvβ3, αvβ5), commonly overexpressed in OSCC, improving delivery efficiency (Wang Z.-Q. et al., 2015; Javid et al., 2024). Ligands like hyaluronic acid targeting CD44 are also widely explored for OSCC (Pornpitchanarong et al., 2020; De Capua et al., 2021).

#### 5.3.2 Antibodies

Nanoparticles conjugated with tumor-specific antibodies provide high selectivity for tumor-associated antigens, enhancing intracellular drug delivery and therapeutic concentration. Additionally, they can engage immune effector cells or block immune checkpoints to stimulate anti-tumor responses (Liu et al., 2021; Tian et al., 2022). EGFR-targeting antibodies, overexpressed in OSCC, are under investigation for nanoparticle-mediated delivery (Cao et al., 2022; Hasan et al., 2025).

#### 5.3.3 Immunomodulators

Functionalizing nanoparticles with immunostimulatory agents such as Toll-like receptor (TLR) ligands or STING agonists promotes dendritic cell activation, antigen presentation, and cytotoxic T cell responses (Zhi et al., 2023; Chen et al., 2024). Targeted delivery to immune cells in the TME or lymph nodes can help reverse local immunosuppression and support combination therapy (SureshBabu et al., 2021; Wells et al., 2024).

These functionalization strategies are particularly valuable in combination therapies. For example, integrating PTT or PDT with ligand- or immunomodulator-functionalized nanoparticles enhances the targeted delivery of photosensitizers or photothermal agents (Li et al., 2023; Shi et al., 2024). This synergy increases local therapeutic efficacy and, when combined with immune activation, can convert immunologically “cold” tumors into “hot” ones, improving response and overcoming resistance (Wu et al., 2021; Shi et al., 2024).

Functionalizing nanoparticles with ligands, antibodies, or immunomodulators holds significant potential for improving drug targeting, immunogenicity, and treatment outcomes in oral cancer (Chen et al., 2024; Wells et al., 2024). Continued research is essential to optimize these systems for clinical translation and large-scale application in OSCC therapy.

## 6 Current state and challenges of DDS in oral cancer

### 6.1 Clinical progress in DDS for oral cancer

Despite growing preclinical evidence supporting drug delivery systems (DDS) for oral squamous cell carcinoma (OSCC), clinical translation remains limited. A notable example is the Phase I/II trial (NCT03502148) evaluating PRV111, a localized, nanoengineered cisplatin delivery patch designed for topical application to oral tumors. PRV111 comprises a dual-layer system: a mucoadhesive chitosan matrix embedded with cisplatin-loaded chitosan particles (CLPs) and an ethyl-cellulose backing to prevent washout by saliva. The system also incorporates a permeation enhancer (PE) to facilitate trans-epithelial transport. Upon moisture exposure, CLPs swell to ∼0.5 μm, larger than vascular pores (2–15 nm), thereby limiting systemic absorption and maximizing local retention. Their cationic surface charge further enhances cellular uptake in tumor tissues (Goldberg et al., 2022).

**FIGURE 5:**
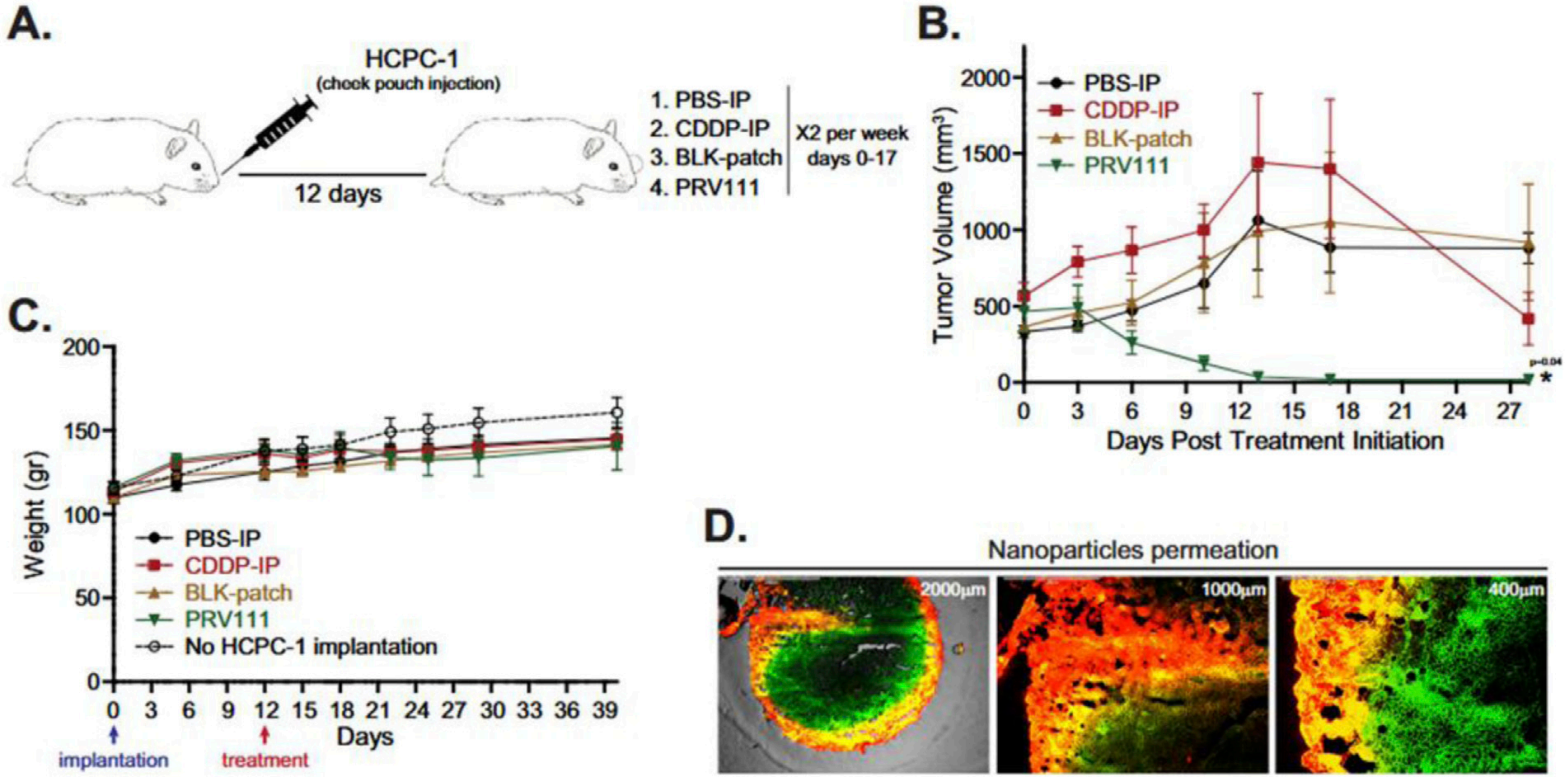
Local application of PRV111 induced robust anti-tumor response in hamster orthotopic oral cancer model. **(A)** Schematic representation of the experiment. **(B)** Golden Syrian hamsters bearing or(thotopic tumors induced by injection of HCPC-1 cell line into the cheek pouch were treated with either PBS-IP, CDDP-IP, BLK-patch or PRV111. Graphs show the average tumor volume for 6 animals per group ± SEM. P-value was performed by a 1-sided Wilcoxon Rank-sum method. Asterisk represents statistical significance between the CDDP-IP and PRV111 groups (p < 0.05). **(C)** Graph shows the average body weights for 6 animals per group ± SEM. Body weights of 5 tumor free animals without treatment were measure along the tumor bearing counterparts (dashed line). **(D)** Representative fluorescence images of tumor sections at indicated magnification taken after treatment of the hamster with PRV111 patch containing chitosan particles labeled with Cy5 (red) and encapsulating FITC (green). Yellow areas display dual-labeling, NPs with encapsulated FITC. Permeation experiment was repeated in 6 tumors (Goldberg et al., 2022).

Preliminary results from the Phase I/II trial (NCT03502148) demonstrated that PRV111 achieved a 69% reduction in tumor volume within 7 days and an 87% overall response rate, without dose-limiting toxicities or severe adverse events. Compared to IV cisplatin, the system yielded ∼259× higher drug concentrations in tumor tissue and ∼182× lower levels in blood, indicating effective local delivery with minimal systemic exposure. Additionally, elevated cisplatin levels in regional lymph nodes and increased tumor-infiltrating lymphocytes (TILs) were observed, suggesting possible immunomodulatory activity alongside cytotoxic effects. These findings highlight PRV111’s potential as a non-invasive, targeted approach for improving therapeutic outcomes in oral cancer (Goldberg et al., 2022). This localized approach could represent a paradigm shift in oral oncology, where non-invasive, effective delivery to cancerous tissues is increasingly desirable (Figure 5) (Goldberg et al., 2022).

Another relevant clinical study investigated NC-6004, a polymeric micelle formulation of cisplatin, in combination with pembrolizumab for patients with head and neck squamous cell carcinoma (HNSCC) resistant to prior platinum-based therapy. Designed to achieve sustained release and selective tumor accumulation via the enhanced permeability and retention (EPR) effect, NC-6004 demonstrated a favorable safety profile with no significant neuro-, oto-, or nephrotoxicity, despite being administered at higher MTD and RP2D than conventional cisplatin. Previous data suggest that this formulation may overcome multidrug resistance, and its use alongside immunotherapy did not impair immune checkpoint inhibitor delivery, supporting its suitability for combination regimens (Osada et al., 2020).

The integration of targeted nanocarrier systems like NC-6004 with immunotherapy offers a promising strategy to circumvent chemoresistance and enhance survival in patients with aggressive, treatment-refractory tumors (Osada et al., 2020).

Recent preclinical studies using 3D oral cancer spheroids have evaluated ZnO and MgO nanoparticles loaded with 5-fluorouracil or cisplatin, showing enhanced bioavailability, cellular uptake, and sustained drug release. These systems improved penetration and distribution within tumor-like structures, potentially reducing resistance and increasing efficacy compared to conventional treatments (Mendoza-Martinez et al., 2023).

Collectively, these clinical and preclinical findings highlight a paradigm shift in oral cancer therapy, where localized, intelligent DDS can achieve high intratumoral concentrations, minimize systemic exposure, and potentially enhance immune activation. As these strategies continue to evolve, their integration into multimodal regimens, including immunotherapy and surgical interventions, may substantially improve outcomes for patients with OSCC.

### 6.2 Challenges: Regulatory, scalability, and personalization in oral cancer treatment

#### 6.2.1 Regulatory hurdles

DDS for oral cancer must meet strict regulatory standards, demonstrating biocompatibility and minimal off-target accumulation—especially important given the proximity of vital oral structures. A thorough understanding of pharmacokinetics and tissue-specific biodistribution is essential to reduce side effects and improve efficacy (Wu et al., 2024). Approval depends on validating the selective accumulation of advanced systems, such as cell membrane-functionalized nanoparticles, in tumor tissues while sparing healthy ones (Shi et al., 2024).

#### 6.2.2 Scalability and manufacturing standards

Scaling DDS for oral cancer is complex due to tumor heterogeneity and the need for high therapeutic specificity. Achieving consistency in particle size, drug loading, and release profiles is critical for targeted delivery and minimizing systemic toxicity (Endo et al., 2013; Goldberg et al., 2022; Peng et al., 2022; Wu et al., 2024). Adhering to Good Manufacturing Practices (GMP) adds further complexity, particularly for patient-specific formulations, posing a barrier to commercialization.

#### 6.2.3 Cost-effectiveness

High costs associated with advanced materials and complex manufacturing processes remain a significant barrier. Although DDS show superior therapeutic potential, their cost often exceeds that of conventional therapies. Demonstrating cost-effectiveness requires comprehensive models that account for long-term savings through better efficacy and reduced adverse effects. Lowering production costs through scalable, GMP-compliant methods is essential for broader clinical adoption (Bosetti and Jones, 2019).

#### 6.2.4 Personalization of DDS

Tailoring DDS to the biological and molecular heterogeneity of OSCC—including HPV-related and tobacco/alcohol-associated subtypes—demands advanced diagnostics and biomarker identification (Shi et al., 2023). Targeted systems designed for specific receptors or altered pathways, such as EGFR or PD-1/PD-L1, could improve precision and efficacy (Maeda et al., 2009; Alexander-Bryant et al., 2017; Peng et al., 2022). Nanoparticles responsive to tumor microenvironmental conditions, such as acidic pH, represent a promising direction (Essawy et al., 2021; Li et al., 2022). Nonetheless, translating these personalized approaches into routine clinical practice remains a technical and economic challenge, particularly in low-resource settings.

## 7 Conclusion

OSCC treatment remains a major challenge due to its aggressive nature, therapeutic resistance, and the significant limitations of conventional approaches (systemic toxicity, poor tumor selectivity, inadequate drug accumulation). These factors lead to suboptimal clinical outcomes, highlighting an unmet need for effective, better-tolerated strategies. Advanced DDS offer innovative solutions by enhancing drug bioavailability, enabling precise targeting, and providing controlled or stimuli-responsive release.

Promising platforms include nanotechnology-based systems (polymeric, inorganic, lipid, exosomes), intelligent hydrogels, and localized therapies like mucoadhesive patches and nanofibers. These DDS are developed to address OSCC-specific challenges such as navigating the dense ECM, responding to unique TME characteristics (e.g., hypoxia, acidic pH, enzymatic activity), targeting overexpressed receptors (e.g., CD44, EGFR), and overcoming physiological barriers (e.g., salivary clearance). Stimuli-responsive systems (e.g., pH, ROS, temperature) and combination therapies integrating DDS with chemotherapy, PDT, PTT, and immunotherapy offer multifaceted approaches to address tumor heterogeneity, bypass drug resistance, and enhance anti-tumor immune responses. Positive preliminary results from clinical trials with novel nanoparticle formulations like PRV111 underscore their translational potential.

Despite these advancements, critical hurdles persist in the clinical translation of novel DDS for OSCC. These include tumor heterogeneity, TME dynamics affecting consistent DDS performance, scalability for reproducible mass production, obtaining robust clinical trial data, and long-term safety profiling. Bridging the gap between innovation and widespread clinical application requires continuous, intensified interdisciplinary collaboration among researchers, pharmaceutical scientists, engineers, clinicians, and regulatory agencies. Addressing these barriers through collaborative efforts, optimization, personalized design, and rigorous clinical validation will enable advanced DDS to redefine the OSCC treatment paradigm, significantly improving therapeutic efficacy, patient outcomes, and quality of life.
